# Inference of the Protokaryotypes of Amniotes and Tetrapods and the Evolutionary Processes of Microchromosomes from Comparative Gene Mapping

**DOI:** 10.1371/journal.pone.0053027

**Published:** 2012-12-31

**Authors:** Yoshinobu Uno, Chizuko Nishida, Hiroshi Tarui, Satoshi Ishishita, Chiyo Takagi, Osamu Nishimura, Junko Ishijima, Hidetoshi Ota, Ayumi Kosaka, Kazumi Matsubara, Yasunori Murakami, Shigeru Kuratani, Naoto Ueno, Kiyokazu Agata, Yoichi Matsuda

**Affiliations:** 1 Department of Applied Molecular Biosciences, Graduate School of Bioagricultural Sciences, Nagoya University, Nagoya, Japan; 2 Department of Biological Sciences, Faculty of Science, Hokkaido University, Sapporo, Japan; 3 Genome Resource and Analysis Subunit, RIKEN Center for Developmental Biology, Kobe, Japan; 4 Division of Morphogenesis, National Institute for Basic Biology, Okazaki, Japan; 5 Department of Biophysics, Graduate School of Science, Kyoto University, Kyoto, Japan; 6 Global COE Program for Evolution and Biodiversity, Graduate School of Science, Kyoto University, Kyoto, Japan; 7 Institute of Natural and Environmental Sciences and Museum of Nature and Human Activities, University of Hyogo, Hyogo, Japan; 8 Laboratory for Evolutionary Morphology, RIKEN Center for Developmental Biology, Kobe, Japan; 9 Department of Basic Biology, School of Life Science, The Graduate University of Advanced Studies (SOKENDAI), Okazaki, Japan; Biodiversity Insitute of Ontario - University of Guelph, Canada

## Abstract

Comparative genome analysis of non-avian reptiles and amphibians provides important clues about the process of genome evolution in tetrapods. However, there is still only limited information available on the genome structures of these organisms. Consequently, the protokaryotypes of amniotes and tetrapods and the evolutionary processes of microchromosomes in tetrapods remain poorly understood. We constructed chromosome maps of functional genes for the Chinese soft-shelled turtle (*Pelodiscus sinensis*), the Siamese crocodile (*Crocodylus siamensis*), and the Western clawed frog (*Xenopus tropicalis*) and compared them with genome and/or chromosome maps of other tetrapod species (salamander, lizard, snake, chicken, and human). This is the first report on the protokaryotypes of amniotes and tetrapods and the evolutionary processes of microchromosomes inferred from comparative genomic analysis of vertebrates, which cover all major non-avian reptilian taxa (Squamata, Crocodilia, Testudines). The eight largest macrochromosomes of the turtle and chicken were equivalent, and 11 linkage groups had also remained intact in the crocodile. Linkage groups of the chicken macrochromosomes were also highly conserved in *X. tropicalis*, two squamates, and the salamander, but not in human. Chicken microchromosomal linkages were conserved in the squamates, which have fewer microchromosomes than chicken, and also in *Xenopus* and the salamander, which both lack microchromosomes; in the latter, the chicken microchromosomal segments have been integrated into macrochromosomes. Our present findings open up the possibility that the ancestral amniotes and tetrapods had at least 10 large genetic linkage groups and many microchromosomes, which corresponded to the chicken macro- and microchromosomes, respectively. The turtle and chicken might retain the microchromosomes of the amniote protokaryotype almost intact. The decrease in number and/or disappearance of microchromosomes by repeated chromosomal fusions probably occurred independently in the amphibian, squamate, crocodilian, and mammalian lineages.

## Introduction

The molecular timescale of vertebrate evolution indicates that synapsids, which developed into mammals, and diapsids, which developed into non-avian reptiles and birds, appeared during the Carboniferous period around 310 million years ago (MYA), after the common ancestor of amniotes (non-avian reptiles, birds, and mammals) diverged from amphibians around 360 MYA [1]–[4]. In general, the karyotypes of non-avian reptiles and birds are characterized by two distinct types of chromosomal component, namely, up to 10 pairs of macrochromosomes and a large number of morphologically indistinguishable microchromosomes. However, crocodilian species, which are the closest living relatives of birds among non-avian reptiles, all lack microchromosomes (Figure S1) [5], [6]. Considering that testudines, which are positioned at the base of Archosauromorpha [7], [8], have a large number of microchromosomes, this phylogenetic pattern suggests either that birds have retained the ancestral state of Archosauromorph karyotypes and microchromosomes disappeared in the crocodilian lineage, or that crocodilians have retained the ancestral state and microchromosomes appeared independently in the bird lineage. There are fewer microchromosomes in squamates than in most testudines and birds, and they are observed neither in the majority of amphibians except for some primitive amphibian species, nor in mammalian or teleost fish species (Figure S1) [6], [9]–[11]. Thus, comparative analysis of reptilian and amphibian genomes is essential for understanding the genome structures that existed at the time of the establishment of ancestral amniotes and tetrapods (amphibians and amniotes), as well as the process of genome evolution in tetrapods, including the origin of microchromosomes.

Genome sequencing projects are now ongoing for many vertebrate species, and the information obtained provides a new perspective on the genome in general and evolution of the chromosomes in vertebrates [12]–[18]. The draft genome assemblies of the green anole (*Anolis carolinensis*) [19] and the Western clawed frog [*Xenopus* (*Silurana*) *tropicalis*] [20] were the first to be reported for non-avian reptiles and amphibians, respectively, and the genetic linkages of these genomes are more highly conserved in chicken chromosomes than those in human chromosomes. Comparisons of genome maps of human, chicken, and teleost fish have suggested that there might be highly conserved linkage homology between the protokaryotypes of amniotes and tetrapods and the chicken karyotype [21]–[24]. This implies that interchromosomal rearrangements (e.g. reciprocal translocations) have occurred rarely in the bird lineage. Comparative genome analysis with the linkage map of the salamander (*Ambystoma mexicanum*/*A. tigrinum*) [25]–[27] also provided evidence that supports this hypothesis. Genome sequencing has now progressed in other non-avian reptile species: two snake species, the garter snake *(Thamnophis sirtalis*) and the Burmese python (*Python molurus bivittatus*) [28], [29], and three crocodilian species, the American alligator (*Alligator mississippiensis*), the saltwater crocodile (*Crocodylus porosus*), and the Indian gharial (*Gavialis gangeticus*) [30]. However, there is still no detailed information on the chromosome maps of Crocodilia and Testudines. In the green anole, many functional genes have been mapped to chromosomes, whereas the homology with chicken chromosomes has not been identified for some microchromosomes [19]. Hence, there are still limits to the available information on the genome structures of non-avian reptiles.

Comparative chromosome mapping of functional genes is a powerful tool to trace the chromosomal rearrangements that have occurred between very distantly related species for which complete genomes have not been sequenced yet. Low rates of evolutionary changes in chromosomes and the low probability of convergence of karyotypes in different lineages make the analysis of karyotypic diversity useful for higher-order phylogenetic studies in vertebrates. Consequently, comparison of chromosome maps between the reptilian species that have many microchromosomes (birds, squamates, testudines) and other tetrapod species without microchromosomes (crocodilians, amphibians, mammals) is a promising approach for understanding the process of karyotypic evolution in amniotes and tetrapods, including the origin of microchromosomes.

Herein, we report high-resolution cytogenetic maps of functional genes for the Chinese soft-shelled turtle (*Pelodiscus sinensis,* 2*n* = 66), the Siamese crocodile (*Crocodylus siamensis*, 2*n* = 30), and the Western clawed frog (*X*. *tropicalis*, 2*n* = 20), and demonstrate highly conserved linkage homology between testudian, crocodilian, and *Xenopus* chromosomes and chicken chromosomes (the Ensembl Chicken Genome Browser, http://www.ensembl.org/Gallus_gallus). Furthermore, we compare our data with: (1) the cytogenetic map of the Japanese four-striped rat snake (*Elaphe quadrivirgata*, 2*n* = 36), which we constructed previously [31], [32]; (2) the genetic linkage map of the salamander (*A. mexicanum/A. tigrinum*) [27]; (3) the genome maps of the green anole (*A. carolinensis*) (the Ensembl Anole Lizard Genome Browser, http://www.ensembl.org/Anolis_carolinensis) [19] and human (the Ensembl Human Genome Browser, http://www.ensembl.org/human), which is considered to have a genomic organization that is similar to the ancestral eutherian karyotype [33]–[36]; and (4) the protokaryotype of teleost fish, which is supposed to contain highly conserved linkage homology with the medaka (*Oryzias latipes*) and the spotted green pufferfish (*Tetraodon nigroviridis*) [17], [24]. In this way, we delineate the protokaryotypes of amniotes and tetrapods, and discuss the evolutionary process of microchromosomes in tetrapods. This is the first comparative genomic analysis of vertebrates to cover all major non-avian reptilian taxa (Squamata, Crocodilia, Testudines).

## Materials and Methods

### Ethics Statement

Animal care and all experimental procedures were approved by the Animal Experiment Committee, Graduate School of Bioagricultural Sciences, Nagoya University (approved no. 2009051401) and Hokkaido University (approved no. CAST04-008 and OAST05-002), and were conducted according to Regulations on Animal Experiments in Nagoya University and Hokkaido University.

### Animals

Embryos of the Chinese soft-shelled turtle (*Pelodiscus sinensis*, Trionychidae, Testudinata) were purchased from a breeding farm in Japan and fibroblast cells were cultured to make chromosome preparations. A two-month-old Siamese crocodile (*Crocodylus siamensis*, Crocodylidae, Crocodilia) of unknown sex, which was derived from captive breeding at the Okinawa Branch of Takada Reptile Farm (associated with Okinawa Zoo, Okinawa Kids Discovery Kingdom Foundation, Okinawa, Japan), was used to construct a brain cDNA library and to generate fibroblast cell cultures. Adult females of the Western clawed frog [*Xenopus* (*Silurana*) *tropicalis*, Pipidae, Anura] of the Yasuda line (Nigerian line established by Dr. R. Grainger) were used to generate fibroblast cell cultures. A crocodile and *Xenopus* were anesthetized with sodium pentobarbital and a solution of 0.1% MS222 (Sigma), respectively, and all efforts were made to minimize suffering.

### cDNA Library, DNA Sequencing, and Homology Search of EST Clones

To construct a cDNA library for *C. siamensis*, poly(A) mRNA was isolated from brain tissue and cloned into the λ uni-ZAP vector (Stratagene) using standard protocols. λ uni-ZAP clones were converted into pBluescript SK(+) clones, and transformed into XL1-Blue bacterial cells (Stratagene). Colonies were picked randomly and transferred into 96-well plates using the ‘Q’ Pix colony picker (GENETIX). The clones were grown overnight and plasmid DNA was prepared using MultiScreen-NA and FB plates (Millipore). Sequencing reactions were performed with dye-labeled dideoxy terminators using the SK primer in accordance with the manufacturer’s protocol (Applied Biosystems), and the nucleotide sequences were determined using an ABI PRISM 3700 DNA Analyzer (Applied Biosystems). Nucleotide sequences were compared against the National Center for Biotechnology Information (NCBI, http://www.ncbi.nlm.nih.gov/) database using the BLASTX program. Individual ESTs were translated in all reading frames and compared against the NCBI “non-redundant” nucleotide and/or peptide sequence database.

For chromosome mapping of *P. sinensis*, a large number of EST clones, which were isolated from cDNA libraries constructed from the brain tissue of a female individual and whole 14-day embryos in our previous study [37], were subjected to a homology search as described above. EST clones of the African clawed frog (*Xenopus laevis*) were used for chromosome mapping of *X. tropicalis*. The genes that were used for chromosome mapping of *X. tropicalis* were chosen on the basis of the chicken chromosome map using a search with the BLASTN programs of Ensembl (http://www.ensembl.org/) and/or NCBI, and we confirmed that these genes used for mapping were each located in different scaffolds of *X. tropicalis* using the genome map of the *X. tropicalis* (the Ensembl Xenopus tropicalis Genome Browser, http://www.ensembl.org/Xenopus_tropicalis). The nucleotide sequences of *X. tropicalis* homologs of the genes were subjected to a search with the BLASTN program of Ensembl, and homologous clones from *X. laevis* were selected from a web data catalogue of the NIBB/NIG/NBRP *Xenopus laevis* EST project (XDB3, http://xenopus.nibb.ac.jp/). All the fragments of *X. laevis* EST clones that were used for chromosome mapping were more than 1.5 kb in size.

### Cell Culture, Chromosome Preparation, and FISH

Cell culture, chromosome preparation, and FISH _(_fluorescence in situ hybridization) were performed as described previously [37]–[40]. Chromosome preparations were made from fibroblast cells taken from embryos of *P. sinensis*, the mesentery of *C. siamensis*, and hearts and kidneys of *X. tropicalis*. The cultured cells were treated with BrdU during late S phase to obtain differential replication banding. Replication-banded chromosomes were obtained by exposing chromosome slides to UV light after staining with Hoechst 33258. EST clones with high E-values were selected as putative reptilian homologs of orthologous chicken genes (Table S1, Table S2), and localized to chromosomes using FISH. EST clones were labeled with biotin-16-dUTP (Roche Diagnostics) by nick translation. After hybridization, the slides were incubated with goat anti-biotin antibodies (Vector Laboratories), and then stained with Alexa Fluor 488 rabbit anti-goat IgG (H+L) conjugate (Invitrogen-Molecular Probes). The chromosome slides were counterstained with 0.75 µg/ml propidium iodide (PI) for observation. FISH images were captured under a fluorescence microscope (Nikon) using B-2A and UV-2A filter sets. Kodak Ektachrome ASA 100 films were used for microphotography. All mapped EST clones (no. FS943139–FS943335) were deposited in the DNA Data Bank of Japan (DDBJ, http://www.ddbj.nig.ac.jp/Welcome.html).

## Results

**Figure 1:**
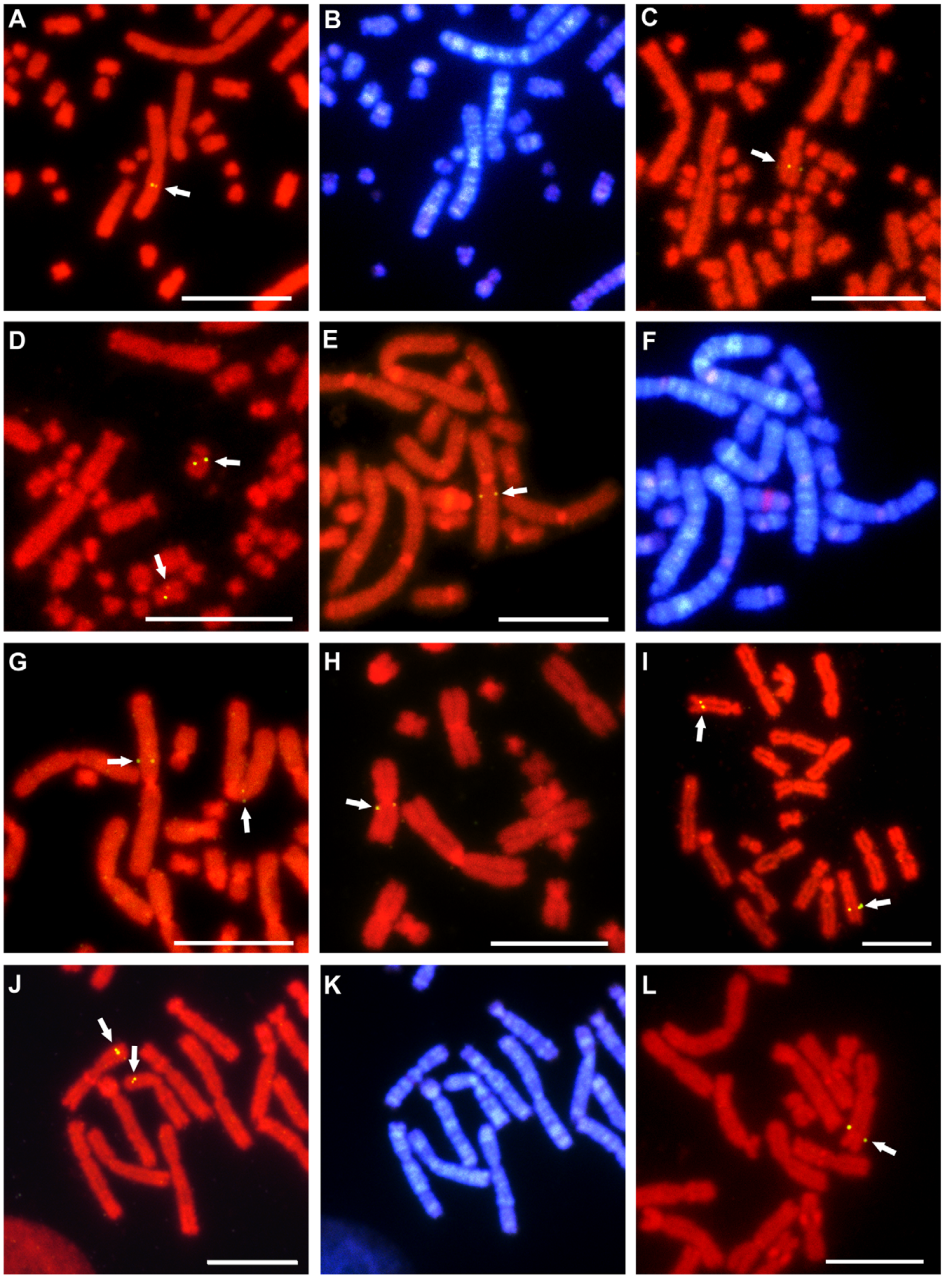
Chromosomal localization of functional genes in *P. sinensis*, *C. siamensis,* and *X. tropicalis*. Localization of cDNA clones to chromosomes of *P. sinensis* (A–D), *C. siamensis* (E–H), and *X. tropicalis* (I–L) by FISH. The *COLEC12* (A), *COQ6* (C), and *SCG2* (D) genes were localized, respectively, to chromosomes 2q and 5q, and the long arms of a submetacentric microchromosomal pair in *P. sinensis*. The *PDCD6* (E), *SON* (G), and *ATP6V1E1* (H) genes were localized, respectively, to chromosomes 3q, 1p, and 4q in *C. siamensis.* The *ACTN1* (I), *USP5* (J), and *LARP4* (L) genes were localized, respectively, to chromosomes 8q, 7p, and 2q in *X. tropicalis*. Hoechst-stained patterns of the PI-stained metaphase spreads in (A), (E), and (J) are shown in (B), (F), and (K), respectively. Scale bars represent 10 µm. Arrows indicate the fluorescence signals.

**Figure 2:**
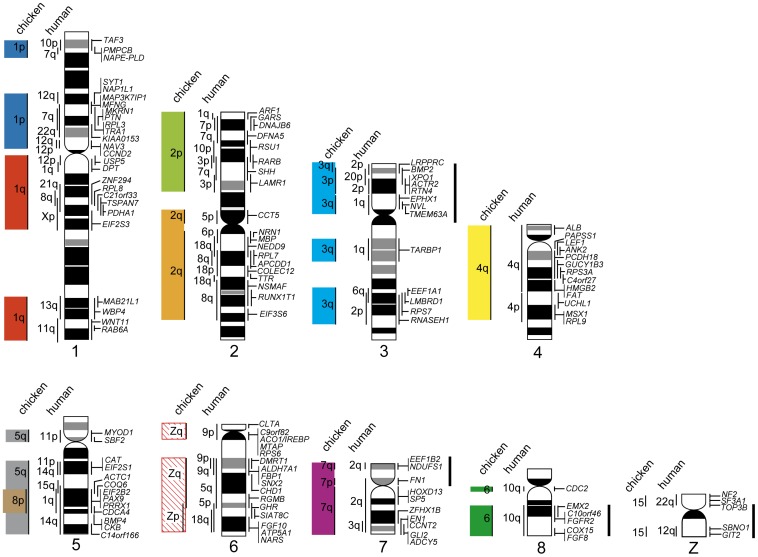
Comparative cytogenetic map of macrochromosomes of the Chinese soft-shelled turtle (*Pelodiscus sinensis*). Homologous chicken and human chromosomes are shown to the left of each turtle chromosome (see Table S1). Genetic linkages that are homologous to chicken macrochromosomal arms and/or macrochromosomes (GGA1p, 1q, 2p, 2q, 3, 4q, and GGA5–8) are represented by 10 differently colored bars, and segments drawn with diagonal lines indicate the chicken Z chromosome. The G-banded ideograms of the turtle chromosomes, which were constructed using Hoechst 33258-stained band patterns obtained by the replication banding method, were taken from our previous report [37]. Solid bars to the right of the turtle chromosomes indicate the chromosomal segments in which intrachromosomal rearrangements occurred that resulted in differences between the turtle and chicken chromosomes.

Previously, we mapped 92 genes to chromosomes of *P. sinensis* [37], [41]–[43]. In the present study, we mapped 70 additional genes using EST clones that were isolated from a cDNA library constructed from *P. sinensis* embryos, and constructed a cytogenetic map with a total of 162 functional genes (Figure 1A–D, Table S1). Of these 162 genes, 116 were localized to the eight largest chromosomes of *P. sinensis*; each of which corresponded to a chicken macrochromosome (Figure 2). All of the 26 genes that were mapped to *P. sinensis* chromosomes (PSI) 1, were located on chicken chromosomes (*Gallus gallus* chromosomes, GGA) 1. Nineteen genes on GGA2, 13 genes on GGA3, 13 genes on GGA4q, 12 genes on GGA5, and 16 genes on GGAZ were localized to PSI2, 3, 4, 5, and 6, respectively, with the exception that *PRRX1* on GGA8 was mapped to PSI5. PSI7 corresponded to GGA7 and PSI8 to GGA 6. The remaining 46 genes, which were localized to the turtle microchromosomes, were also located on chicken microchromosomes (GGA9–15, 17–23, 26, 28), with the exception of five genes on the short arm of chicken chromosome 4 (GGA4p). GGA4p was derived from a microchromosome that fused with the acrocentric chromosome 4 of the avian common ancestor [14]. The turtle Z micro-sex chromosome corresponds to GGA15 [43]. Comparison of the gene orders between the turtle and chicken chromosomes revealed the presence of intrachromosomal rearrangements (e.g. inversions) in PSI3p, 7p, 8q, and Z, but no interchromosomal rearrangements were found (Figure S2).

**Figure 3:**
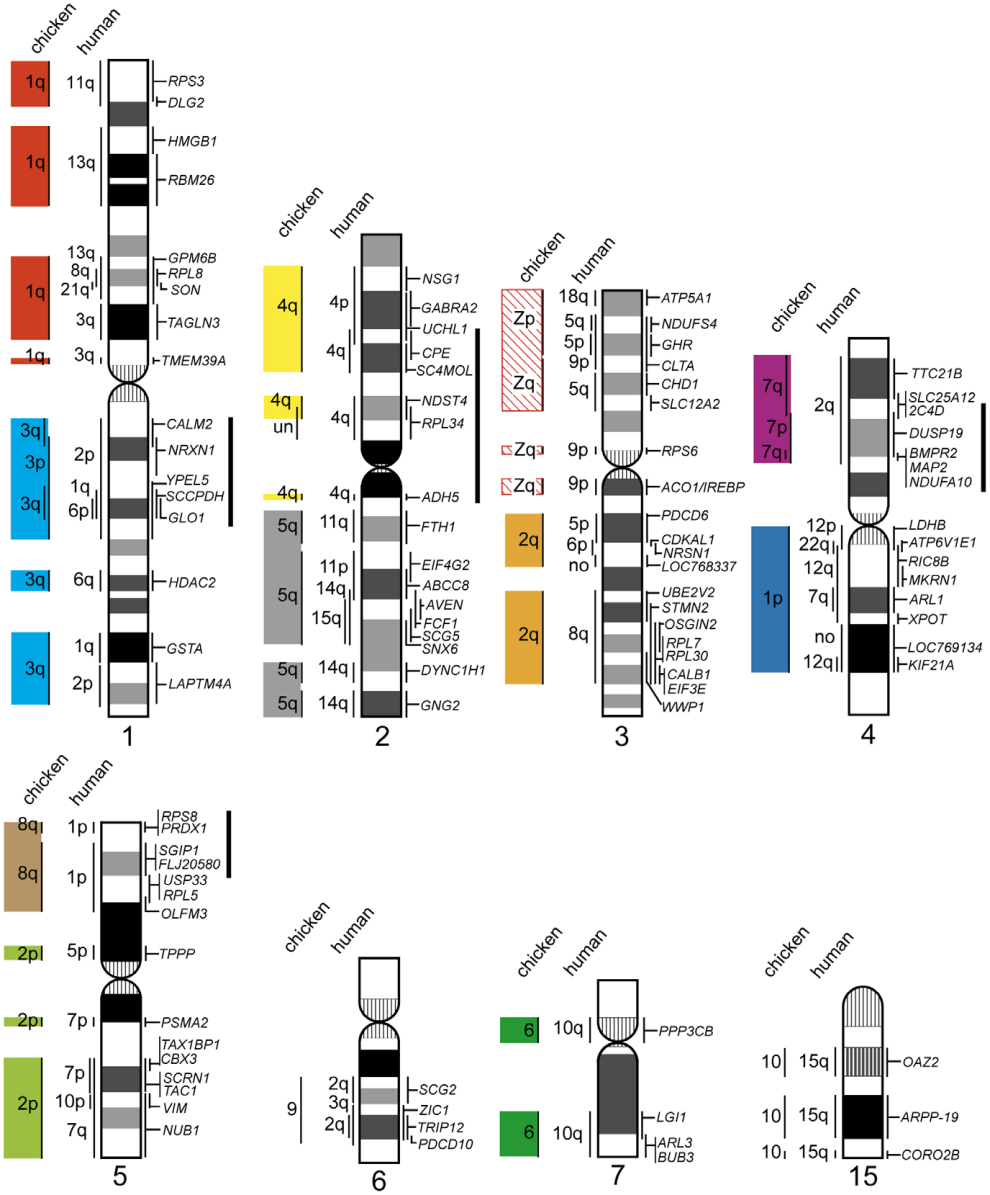
Comparative cytogenetic map of the eight largest chromosomes of the Siamese crocodile (*Crocodylus siamensis*). Homologous chicken and human chromosomes are shown to the left of each crocodile chromosome (see Table S2). Genetic linkages of chicken macrochromosomal arms and/or macrochromosomes are represented by the same colored bars as those in Figure 2. The G-banded ideograms of the crocodile chromosomes were constructed in the present study by the same method as that used for the turtle chromosomes [37]. Solid bars to the right of the crocodile chromosomes indicate the chromosomal segments in which intrachromosomal rearrangements occurred between the crocodile and chicken. un, chromosomal location is unknown in chicken. no, no homologs were found.

All the 23 extant crocodilian species (2*n* = 30–42) lack microchromosomes, and most of the species (16 species), which include *C. siamensis* [2*n* = 30, fundamental number (NF) = 58], have 30–34 chromosome pairs (Figure S1) [6]. Thus, *C*. *siamensis* is a good model species for investigating the process of microchromosomal evolution in amniotes. We mapped 131 genes to the crocodile chromosomes (*Crocodylus siamensis* chromosomes, CSI) using EST clones that were isolated from a brain cDNA library of *C. siamensis* (Table S2). Ninety-five out of the 131 genes were localized to the eight largest chromosomes (CSI1–7 and 15) (Figure 1E–H, Figure 3). Highly conserved linkage homology was observed between the crocodile and chicken chromosomes: CSI1p, 1q, 2p, 2q, 3p, 3q, 4p, 4q, 5p, and 5q corresponded exactly to GGA1q, 3, 4q, 5, Z, 2q, 7, 1p, 8q, and 2p, respectively. Furthermore, CSI6q corresponded to GGA9, CSI7 to GGA6, and CSI15 to GGA10. No interchromosomal rearrangements were found between the two species; however, there were intrachromosomal rearrangements in at least four chromosomes (CSI1, 2, 4, and 5) (Figure S3). Another remarkable result was that the regions that were homologous to GGA4q, Z, and 2p on CSI2p, 3p, and 5q, respectively, traversed the centromeres to include sections of the other arms of the same chromosomes. This finding suggested that small pericentric inversions or repositioning of the centromere occurred after centromeric fusions between acrocentric chromosomes; however, no data were obtained to confirm these hypotheses in the present study. Three genes that are located on GGA4p and 33 genes that are located on chicken microchromosomes (GGA11–15, 17–21, 23, 26, 28) were all localized to small macrochromosomes (CSI8–14) that were smaller than CSI15.

**Figure 4:**
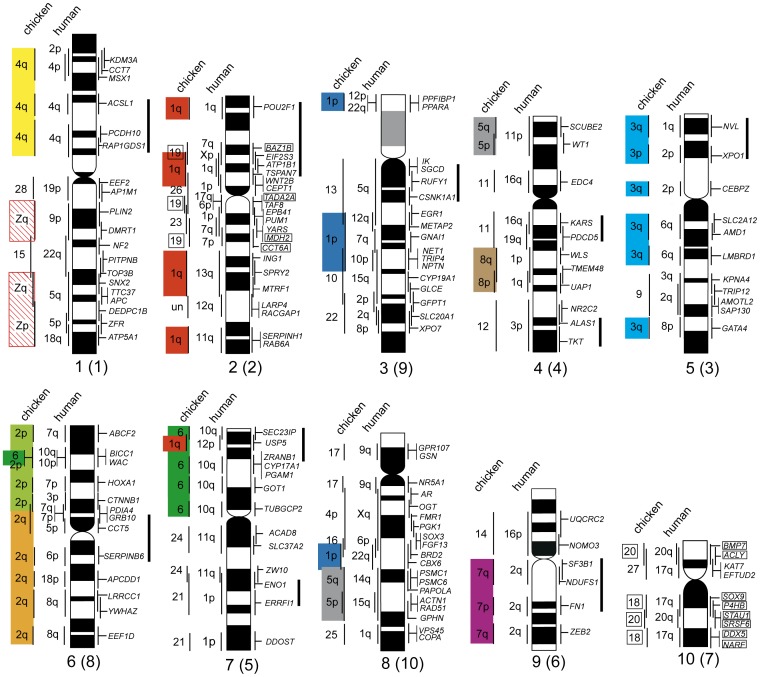
Comparative cytogenetic map of *Xenopus* (*Silurana*) *tropicalis*. Homologous chicken and human chromosomes are shown to the left of each *X. tropicalis* chromosome (see Table S3). Genetic linkages of chicken macrochromosomal arms and/or macrochromosomes are represented by the same colored bars as those in Figure 2. The G-banded ideograms of *X. tropicalis* chromosomes were taken from our previous report [40]. Chromosomes are ordered in accordance with Hellsten et al. [20]. Numbers in parentheses indicate chromosome numbers from our previous report [40]. Solid bars to the right of the *X. tropicalis* chromosomes indicate the chromosomal segments in which intrachromosomal rearrangements occurred between *X. tropicalis* and chicken. Gene symbols and chicken chromosome numbers enclosed in boxes indicate the chromosomal segments that corresponded to chicken microchromosomes in which intrachromosomal rearrangements had occurred. un, chromosomal location is unknown.

Previously, we mapped eight genes to *X. tropicalis* chromosomes (XTR) [40] and, in the present study, we constructed a cytogenetic map of *X. tropicalis* that contained a total of 140 genes (homologs of 77 macrochromosomal and 63 microchromosomal genes in chicken) using EST clones of *X. laevis* (Figure 1I–L, Figure 4, Table S3). Linkage homology was much higher between *X. tropicalis* and chicken chromosomes than between chicken and human chromosomes [20]. The 10 genetic linkages of chicken macrochromosomes (GGA1p, 1q, 2p, 2q, 3, 4q, GGA6–8, Z) have been retained almost intact in *X. tropicalis*. Most of the genes located on GGA1p, 1q, and 6 were mapped to XTR3, 2, and 7p, respectively. However, single genes from these chromosomes, namely, *CBX6* on GGA1p, *USP5* on GGA1q, and *BICC1* on GGA6, were located on XTR8q, 7p, and 6p, respectively, which indicated that small translocations might have occurred between XTR2 and 7p, between XTR3 and 8q, and between XTR7p and 6p. The linkage group of GGA5 was separated into two chromosomal segments on XTR4p and 8q, respectively. Four genetic linkage groups on GGA1p, 1q, 3, and Z, which were mapped to XTR3, 2, 5, and 1q, respectively, were interrupted on the *X. tropicalis* chromosomes by insertions of chromosomal segments that corresponded to chicken microchromosomes. Sixty-one genes of chicken microchromosomes (GGA9–28) were localized to *X. tropicalis* chromosomes, with the exception of XTR6. The genetic linkages of 19 chicken microchromosomes (GGA9–15, 17–28) and GGA4p have been conserved in *X. tropicalis* chromosomes (Figure 4, Figure S4, Table S3); two or more genes were localized in each of these chicken microchromosomes. These linkage groups have been integrated into chromosomes or fused tandemly to chromosomes in *X. tropicalis*. Four entire arms of three *X. tropicalis* chromosomes (XTR7q, 9p, 10p, and 10q) were found to be composed of chromosome fragments that corresponded to chicken microchromosomes. Intrachromosomal rearrangements between *Xenopus* and chicken were found in XTR1p, 2p, 3q, 4q, 5p, 6, 7p, 7q, and 9q and in the regions that were homologous to GGA19 on XTR2 and to GGA18 and 20 on XTR10 (Figure 4, Figure S4). These highly conserved linkage homologies between *X. tropicalis* chromosomes and chicken macro- and microchromosomes suggest that few interchromosomal rearrangements occurred during the process of evolution from amphibians to amniotes [20], [25]–[27].

## Discussion

**Figure 5:**
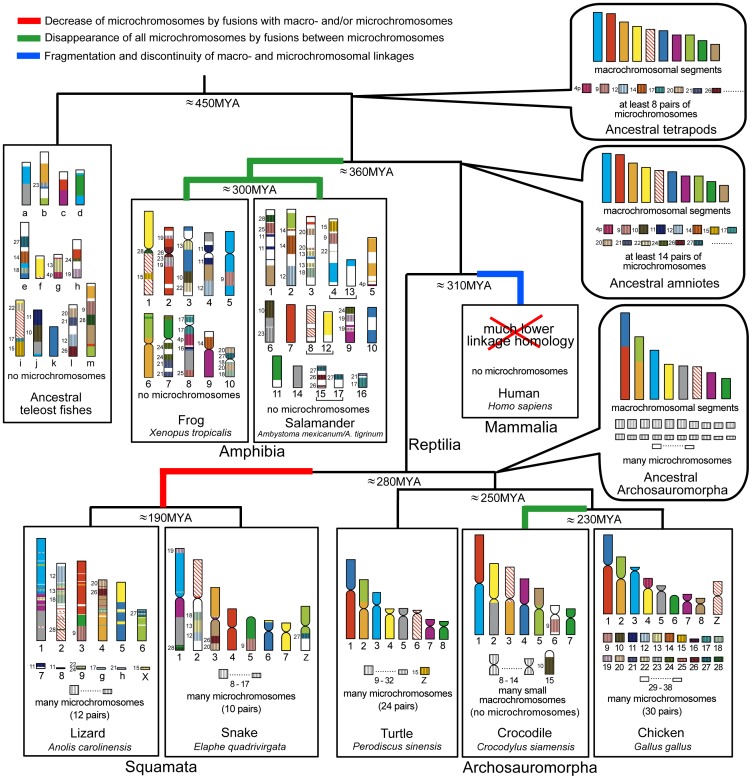
Schematic representation of the protokaryotypes of amniotes and tetrapods and their evolutionary processes. The schematic diagrams of the vertebrate chromosomes are modified from the genome and/or chromosome maps of *Ambystoma mexicanum*/*A. tigrinum* [27], the green anole (*Anolis carolinensis*) (the Ensembl Anole Lizard Genome Browser, http://www.ensembl.org/Anolis_carolinensis) [19], the Japanese four-striped rat snake (*Elaphe quadrivirgata*) [31], [32], and chicken (the Ensembl Chicken Genome Browser, http://www.ensembl.org/Gallus_gallus), and the protokaryotype of teleost fishes [17], [24]. Genetic linkages that are homologous to chicken macrochromosomal arms and/or macrochromosomes (GGA1p, 1q, 2p, 2q, 3, 4q, and GGA5–8) and microchromosomes (GGA4p and GGA9–28) are represented by 10 and 21 differently colored bars, respectively, and segments drawn with diagonal lines indicate the chicken Z chromosome. The chromosome numbers of the chicken microchromosomes are shown to the left of the chromosomes for the reptilian and amphibian species and the ancestral amniote, tetrapod, and teleost fish. The ancestral amniotes and tetrapods had at least 10 large genetic linkage groups, which corresponded to chicken macrochromosomes. At least 14 and eight pairs of microchromosomes, which were homologous to chicken microchromosomes, were also contained in the protokaryotypes of amniotes and tetrapods, respectively. The macrochromosomal genetic linkages of tetrapods have been highly conserved in amphibians, non-avian reptiles, and birds for over 360 million years. Fusions between macro- and microchromosomes and/or between microchromosomes occurred independently in the amphibian, squamate, crocodilian, and mammalian lineages, although the fusions occurred very rarely or less frequently in the testudian and avian lineages. Homologies with chicken macro- and microchromosomal linkage groups are much lower in human [22]–[24]. In the salamander, linkage 4 and 13, linkage 8 and 12, and linkage 15 and 17 are each contained in the same linkage [26], [27], [49] (Figure S5). The divergence times are cited from Hedges et al. [2] and Benton & Donoghue [3]. MYA, million years ago.

Comparative gene mapping for the Chinese soft-shelled turtle (*_P. sinensis_*_)_ revealed that the macro- and microchromosomes of this turtle are true counterparts of those of chicken. This extensive homology between the turtle and chicken chromosomes suggests that the ancestral karyotype of Archosauromorpha consisted of two major components, namely, at least eight pairs of macrochromosomes and many indistinguishable microchromosomes. These components are homologous to the chicken macro- and microchromosomes, respectively. This protokaryotype has been highly conserved for more than 250 million years since the lineage diverged from Lepidosauromorpha (Figure 5) [1]–[4], [7], [8].

In the Siamese crocodile (*C. siamensis*), the genetic linkages of the macrochromosomes have also been conserved in blocks on chromosome arms that correspond to chicken macrochromosomal arms and/or entire macrochromosomes. However, the five largest bi-armed chromosomes of the crocodile are formed from combinations of chromosome arms that differ from the chicken karyotype. In addition, the chicken microchromosomal genes that were used for chromosome mapping were all localized to small macrochromosomes of the crocodile. These results suggest that the Siamese crocodile karyotype resulted from two events that occurred in the crocodilian lineage: (i) centric fissions of bi-armed macrochromosomes in the ancestral Archosauromorph karyotype followed by centric fusions between the resultant acrocentric macrochromosomes; and (ii) repeated fusions between microchromosomes, which resulted in the disappearance of microchromosomes and the appearance of a large number of small macrochromosomes.

Eleven linkage groups, which correspond to chicken macrochromosomes GGA1p, 1q, 2p, 2q, 3, 4q, GGA5–8, and Z, are highly conserved in the green anole and the Japanese four-striped rat snake [19], [29], [32] (Figure 5). In our previous study, the butterfly lizard (*Leiolepis reevesii rubritaeniata*, 2*n* = 36) showed highly conserved linkage homologies with the snake chromosomes [44]. Ten of the 11 macrochromosomal linkage groups, with the exception of GGA5, were also conserved in *X. tropicali*s and the salamander (Figure S5A–K), whereas their linkage homologies are much lower in human chromosomes [22]–[24]. Collectively, these results suggest that the ancestral karyotype of amniotes might have contained at least 10 large genetic linkage groups that were each homologous to GGA1p, 1q, 2p, 2q, 3, 4q, GGA6–8, and Z. In contrast, in the mammalian lineage, chromosomal rearrangements (reciprocal translocations, fusions, fissions, inversions, etc.) have occurred at high frequency, which has led to the fragmentation and discontinuity of macro- and microchromosomal linkages of the ancestral amniote karyotype [22]–[24]. In the protokaryotype of teleost fish, which was proposed by Kasahara et al. [17] and Nakatani et al. [24], eight of the 11 chicken macrochromosomal linkages (namely, GGA1p, 1q, 2p, 2q, 3, 4q, 5, Z) are divided into two, or more than two, genetic linkages (Figure S5A–K), which indicates much lower homology with chicken macrochromosomes in the ancestral teleost fish.

**Figure 6:**
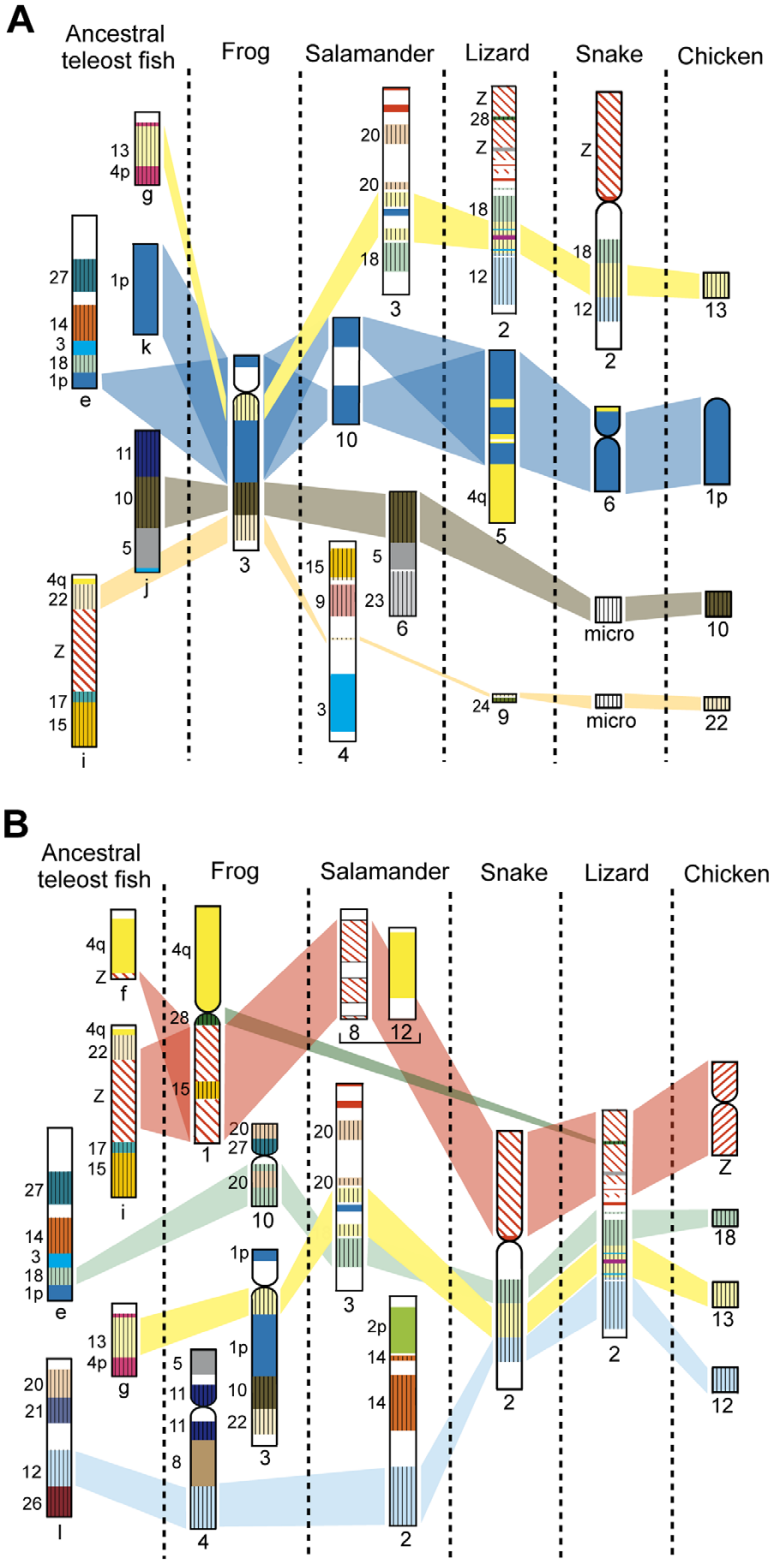
Comparison of chromosomal locations of chicken microchromosomal linkages among vertebrate species. The chromosomal locations of chicken microchromosomal linkages on *X. tropicalis* chromosome 3 (A) and *E. quadrivirgata* chromosome 2 (B) are compared among four tetrapod species, *X. tropicalis*, salamander (*A. mexicanum/A. tigrinum*), lizard (*A. carolinensis*), and snake (*E. quadrivirgata*), and ancestral teleost fish. Genetic linkages of chicken macro- and microchromosomes are represented by the same colored bars as those in Figure 5, and each conserved genetic linkage was defined when two or more genes were located on each of chicken chromosomes. The chromosome numbers of the chicken microchromosomes are shown to the left of each chromosome. Information on the genetic linkages for the ancestral teleost fish, salamander, snake, lizard, and chicken was taken from Kasahara et al. [17] and Nakatani et al. [24], Voss et al. [27], Matsubara et al. [31], [32], Alföldi et al. [19] and the Ensembl Anole Lizard Genome Browser (http://www.ensembl.org/Anolis_carolinensis), and the Ensembl Chicken Genome Browser (http://www.ensembl.org/Gallus_gallus), respectively. The lizard chromosome that is homologous to GGA10 has not been identified yet. The genetic linkages of GGA10, 13, and 22 on *X. tropicalis* chromosome 3 and GGA12, 13, and 18 on snake chromosome 2 were localized to nonhomologous chromosomes in the other species, except for GGA13 and GGA18 on salamander chromosome 3. The salamander linkage 8 and 12 were contained in the same linkage group [26], [27], [49].

Squamates have far fewer microchromosomes than chicken and the Chinese soft-shelled turtle. All microchromosomes of the Japanese four-striped rat snake are homologous to chicken microchromosomes. However, approximately half the linkage groups that are homologous to the chicken microchromosomes are integrated into macrochromosomes in this species [31], [32] (Figure 5). The genetic linkages of chicken microchromosomes were also highly conserved in *X. tropicalis*, as well as in the green anole, salamander, and ancestral teleost fish (Figure S5L–C’), whereas these linkages are hardly conserved in human [22]–[24]. However, there is little homology with respect to their sites of integration among amphibians, squamates, and the ancestral teleost fish. For instance, *X. tropicalis* chromosome 3 (XTR3) contains three chicken microchromosomal linkages (GGA10, 13, and 22), whereas these linkage groups are localized separately to nonhomologous chromosomes in the salamander (linkage groups 3, 4, and 6), the snake (chromosome 2 and two pairs of microchromosomes), the green anole (chromosome 2 and a pair of microchromosomes, but no homologous region of GGA10 has been identified), and the ancestral teleost fish (linkage groups g, i, and j) (Figure 6A). Chromosome 2 of the snake and lizard contains three linkage groups that are homologous to GGA12, 13, and 18, whereas these linkage groups are localized to nonhomologous chromosomes in *Xenopus* (XTR3, 4, and 10) and the ancestral teleost fish (linkage groups e, g, and l). However, in the salamander, the linkages of GGA13 and 18 are integrated into the same linkage group (linkage group 3), as in the snake and lizard (Figure 6B). Consequently, no homologies were found for the sites of integration of 14 chicken microchromosomal linkage groups (GGA4p, 9, 10, 11, 12, 14, 15, 17, 20, 21, 22, 24, 26, and 27) between amphibians and squamates (Figure S5L–C’). This result indicates the possibility that no chromosomal fusions occurred between the 14 chicken microchromosomal linkages and the other chicken macro- and microchromosomal linkages after the common ancestor of amniotes diverged from Amphibia. Among these 14 chicken microchromosomal linkage groups, linkage homologies were found for the integration sites of six linkage groups (corresponding to GGA10, 11, 15, 22, 24, and 27) between the ancestral teleost fish and amphibians (Figure S5L–C’). This finding suggested that the other eight chicken microchromosomal linkages (GGA 4p, 9, 12, 14, 17, 20, 21, and 26) might have been existed as microchromosomes in the ancestral karyotype of tetrapods before amphibians first appeared.

Through comparative analysis of genome maps among human, chicken, and teleost fish, Nakatani et al. [24] also speculated on the presence of 12 pairs of microchromosomes (corresponding to GGA4p, 11, 12, 15, 19, 20, 21, 22, 23, 24, 27, and 28) in ancestral amniotes (see Figure 4 and Table S3 in Nakatani et al. [24]). Voss et al. [27] suggested, by comparing maps among *Xenopus*, salamander, and chicken, that at least nine chicken microchromosomal linkages (GGA14, 15, 17, 19, 20, 21, 23, 24, and 28) existed in the karyotype of ancestral tetrapods (see Table 1 in Voss et al. [27]). In the present study, comparison of genome and/or chromosome maps among five major tetrapod groups (Amphibia, Squamata, Crocodilia, Testudines, and Aves) and the protokaryotype of teleost fish suggested strongly that 14 chicken microchromosomal linkages (GGA4p, 9, 10, 11, 12, 14, 15, 17, 20, 21, 22, 24, 26, and 27) probably existed in the ancestral karyotype of amniotes. This differs slightly from the group of linkages proposed by Nakatani et al. [24]. Our data suggest the possibility that eight microchromosomal linkages (GGA4p, 9, 12, 14, 17, 20, 21, and 26) that were found in ancestral tetrapods were each retained intact as one pair of microchromosomes in ancestral amniotes (Figure 5). After divergence from the ancestral tetrapod, at least two insertion events (GGA9 on XTR5, GGA26 on XTR2) and many tandem fusion events of microchromosomes (GGA4p, 12, 14, 17, 20 and 21) occurred in *X. tropicalis* lineage. In the salamander lineage, many tandem fusion events also occurred; however no evidences of insertion events of microchromosomes have been detected in the salamander. In squamates, the microchromosomes that correspond to chicken microchromosomes in the protokaryotype of amniotes might have been integrated frequently into macrochromosomes by fusions between macro- and microchromosomes, while no insertions of microchromosomes into macrochromosomes have occurred (except for GGA28 to GGAZ on the lizard chromosome 2). In the crocodilian lineage, all microchromosomes disappeared by fusion between microchromosomes. In mammals, platypus (*Ornithorhynchus anatinus*), which belongs to Monotremata, has many small chromosomes. However, there is little homology between chicken microchromosomes and the small chromosomes of platypus [45], which suggests that the microchromosomes of ancestral amniotes disappeared in the mammalian lineage before Monotremata diverged around 170 MYA [2], [3].

Microchromosomes are observed predominantly in species that are related closely to the ancestral tetrapods, for example, the Comoros coelacanth (*Latimeria chalumnae,* Coelacanthiformes, 2*n* = 48, which contains eight pairs of microchromosomes) [46] and the Australian lungfish (*Neoceratodus forsteri*, Ceratodontiformes, 2*n* = 54, 10 microchromosome pairs) [47]. They are also observed in the phylogenetically ancestral lineage of urodeles and anurans: Hynobiidae and Cryptobranchidae (2*n* = 56–78, more than 15 microchromosome pairs in most species [10]) and the tailed frog (*Ascaphus truei*, Leiopelmatidae, 2*n* = 46, 13 microchromosome pairs) [9]. The presence of microchromosomes in these species might have some connection with the possibility that the karyotypes of ancestral amniotes and tetrapods contained microchromosomes, as supposed in the present study as well as by Nakatani et al. [24] and Voss et al. [27]. Sequencing of the entire chicken genome has revealed that there are distinct structural differences between chicken macro- and microchromosomes in terms of recombination rate, G+C and CpG contents, gene density, density of repeat sequences, rate of synonymous nucleotide substitutions, and other features [14]. We also found that the genes of the microchromosomes in the Chinese soft-shelled turtle [41] and the Japanese four-striped rat snake [32] are more GC-rich than those of the macrochromosomes, as is the case in chicken. However, the microchromosomes of the green anole do not exhibit a higher G+C content than the macrochromosomes, which indicates that there is no striking bias with respect to interchromosomal G+C content in this species [19], [48]. Comparative chromosome mapping in species that are related closely to ancestral tetrapods (the coelacanth and Australian lungfish) and the primitive urodeles and anurans that have many microchromosomes will enable us to elucidate the origins of microchromosomes and the process of genomic compartmentalization between macro- and microchromosomes in amniotes.

## Supporting Information

Figure S1 **Giemsa-stained karyotypes of chicken, turtle, crocodile, snake, and frog.** Giemsa-stained karyotypes of (A) chicken (*Gallus gallus*, 2*n* = 78), (B) the Chinese soft-shelled turtle (*Pelodiscus sinensis*, 2*n* = 66), (C) the Siamese crocodile (*Crocodylus siamensis*, 2*n* = 30), (D) the Japanese four-striped rat snake (*Elaphe quadrivirgata*, 2*n* = 36), and (E) the Western clawed frog [*Xenopus* (*Silurana*) *tropicalis*, 2*n* = 20]. The chromosomes of *X. tropicalis* are ordered in accordance with Hellsten et al. [20], and numbers in parentheses indicate chromosome numbers from our previous report [40]. Scale bar represents 10 µm.
(TIF)

Figure S2 **Comparative map of chicken homologs of** ***P. sinensis*** **genes.** Chromosomal locations of chicken homologs were identified using the BLASTN programs of Ensembl and/or NCBI (retrieved in March 2012). Horizontal bars inside the chromosomes represent the locations of centromeres, which are defined as gaps in the golden path. Solid vertical bars to the right of chromosomes indicate the chromosomal regions in which intrachromosomal rearrangements occurred that resulted in differences between chicken and turtle.
(TIF)

Figure S3 **Comparative map of chicken homologs of** ***C. siamensis*** **genes.** Chromosomal locations of chicken homologs were identified using the BLASTN programs of Ensembl and/or NCBI (retrieved in March 2012). Horizontal bars inside chromosomes represent the locations of centromeres. Solid vertical bars to the right of chromosomes indicate the chromosomal regions in which intrachromosomal rearrangements occurred between chicken and crocodile.
(TIF)

Figure S4 **Comparative map of chicken homologs of** ***Xenopus*** **genes.** Chromosomal locations of chicken homologs were identified using the BLASTN programs of Ensembl and/or NCBI (retrieved in March 2012). Horizontal bars inside chromosomes represent the locations of centromeres. Solid vertical bars to the right of chromosomes indicate the chromosomal regions in which intrachromosomal rearrangements occurred between chicken and *X. tropicalis*.
(TIF)

Figure S5 **Comparison of chromosomal locations of chicken chromosomal linkages among frog, salamander, lizard, snake, and ancestral teleost fish.** Comparison of chromosomal locations of chicken macrochromosomal linkages (GGA1p, 1q, 2p, 2q, 3, 4q, GGA5–8, and Z) (A–K) and microchromosomal linkages (GGA4p, 9–15, 17–22, 24, and 26–28) (L–C’) among the frog (*X. tropicalis*), salamander (*Ambystoma mexicanum/A. tigrinum*), lizard (*Anolis carolinensis*), snake (*E. quadrivirgata*), and ancestral teleost fish. Genetic linkages of chicken macro- and microchromosomes are represented by the same colored bars as those in Figure 5, and each conserved genetic linkage was defined when two or more genes were located on each of chicken chromosomes. The numbers of the homologous chicken chromosomes are shown to the left of the chromosomes. Eleven linkages of chicken macrochromosomes (1p, 1q, 2p, 2q, 3, 4q, GGA5–8, and Z) have been highly conserved in two squamates, and 10 of the 11 macrochromosomal linkages, with the exception of GGA5, were also conserved in *X. tropicali*s and the salamander. In contrast, the linkage homology was found to be much lower between chicken macrochromosomes and the ancestral teleost fish chromosomes. Five chicken microchromosomal linkages (GGA9, 12, 20, 26, and 27) were each integrated into chromosomes that were nonhomologous between amphibians and squamates. However, linkage homologies with respect to sites of integration were found for four linkage groups (corresponding to GGA13, 18, 19, and 28) between amphibians and squamates. The genetic linkages of GGA13 and GGA18 were co-located on single chromosomes of the salamander (chromosome 3) and the two squamates (chromosome 2). In addition, the genetic linkages of GGA19 and GGA7 were co-located on salamander chromosome 9 and snake chromosome 1, and those of GGA28 and GGAZ on *Xenopus* chromosome 1 and lizard chromosome 2. Nine genetic linkages (corresponding to GGA4p, 10, 11, 14, 15, 17, 21, 22, and 24) have remained as microchromosomes in squamates. There were no linkage homologies with respect to sites of integration for eight genetic linkages (GGA4p, 9, 12, 14, 17, 20, 21, 26) out of the 14 chicken microchromosomal linkages (GGA4p, 9, 10, 11, 12, 14, 15, 17, 20, 21, 22, 24, 26, and 27) between the ancestral teleost fish and amphibians. Three genetic linkage groups (GGA16, 23, and 25) were excluded from this Figure because the chromosomal regions homologous to these linkages were not identified in the chromosome maps of the lizard and snake [19], [31], [32]. In the salamander, linkage 4 and 13, linkage 8 and 12, and linkage 15 and 17 are each contained in the same linkage [26], [27], [49].
(TIF)

Table S1 List of 162 genes that were localized to chromosomes of *P. sinensis.*
(DOC)

Table S2 List of 131 genes that were localized to chromosomes of *C. siamensis.*
(DOC)

Table S3 List of 140 genes that were localized to chromosomes of *X. tropicalis.*
(DOC)
