# Supplementary figures and images for: Inference of the Protokaryotypes of Amniotes and Tetrapods and the Evolutionary Processes of Microchromosomes from Comparative Gene Mapping
Source: PLoS One. 2012 Dec 31;7(12):e53027. doi: 10.1371/journal.pone.0053027 (PMC3534110; doi:10.1371/journal.pone.0053027)

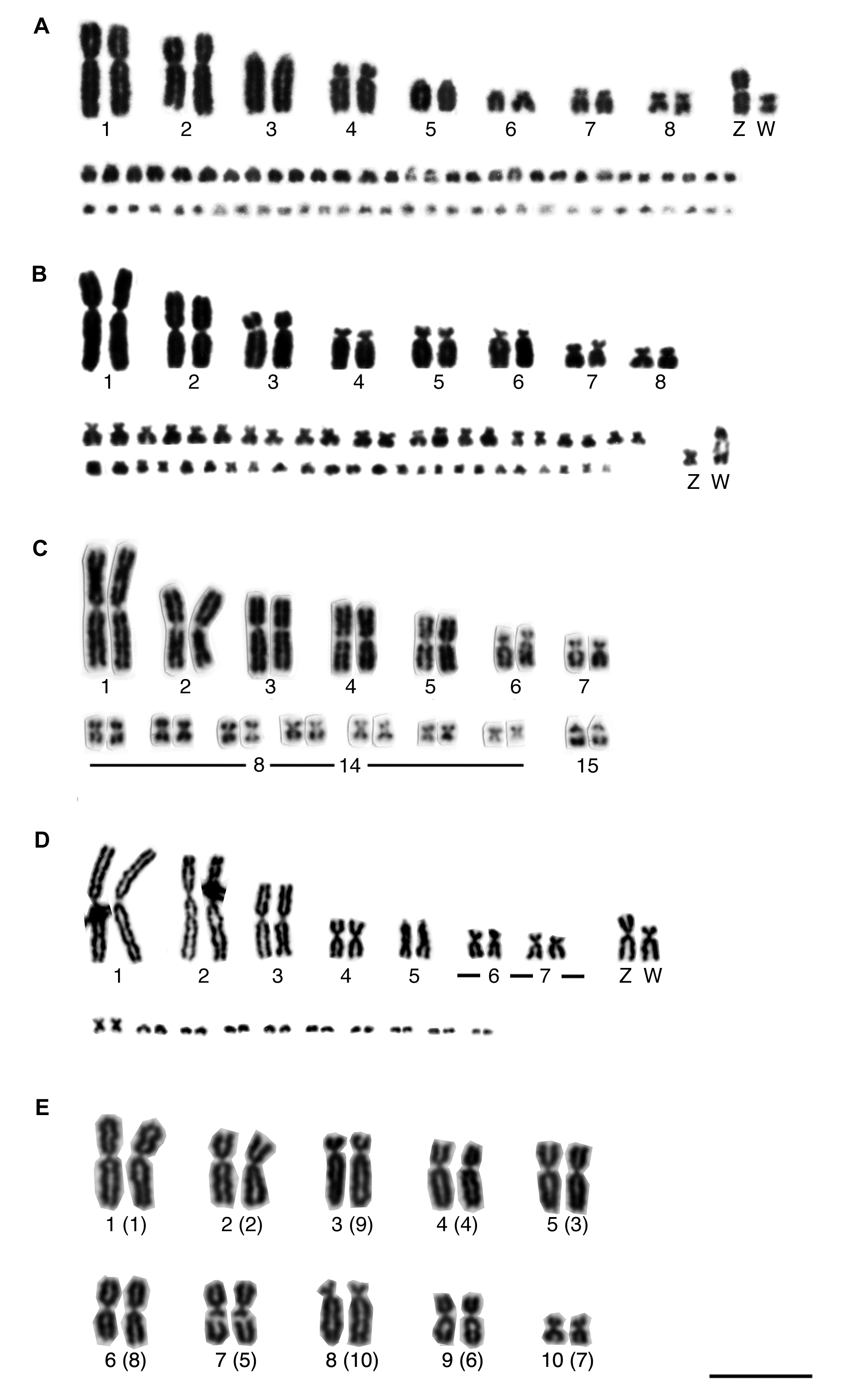

Supplement: Figure S1 — Giemsa-stained karyotypes of chicken, turtle, crocodile, snake, and frog. Giemsa-stained karyotypes of (A) chicken (Gallus gallus, 2n = 78), (B) the Chinese soft-shelled turtle (Pelodiscus sinensis, 2n = 66), (C) the Siamese crocodile (Crocodylus siamensis, 2n = 30), (D) the Japanese four-striped rat snake (Elaphe quadrivirgata, 2n = 36), and (E) the Western clawed frog [Xenopus (Silurana) tropicalis, 2n = 20]. The chromosomes of X. tropicalis are ordered in accordance with Hellsten et al. [20], and numbers in parentheses indicate chromosome numbers from our previous report [40]. Scale bar represents 10 µm. (TIF) [file pone.0053027.s001.tif]

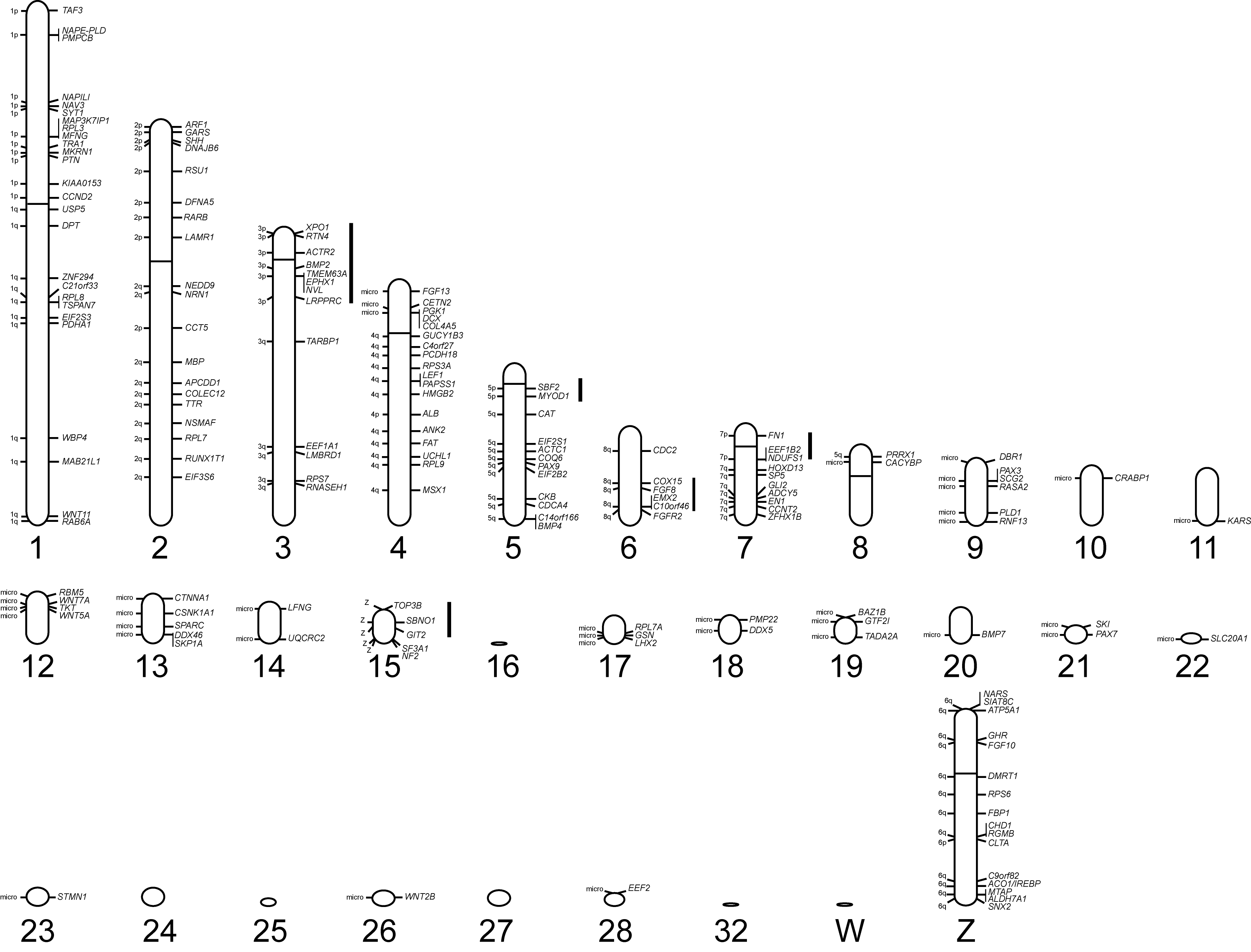

Supplement: Figure S2 — Comparative map of chicken homologs of P. sinensis genes. Chromosomal locations of chicken homologs were identified using the BLASTN programs of Ensembl and/or NCBI (retrieved in March 2012). Horizontal bars inside the chromosomes represent the locations of centromeres, which are defined as gaps in the golden path. Solid vertical bars to the right of chromosomes indicate the chromosomal regions in which intrachromosomal rearrangements occurred that resulted in differences between chicken and turtle. (TIF) [file pone.0053027.s002.tif]

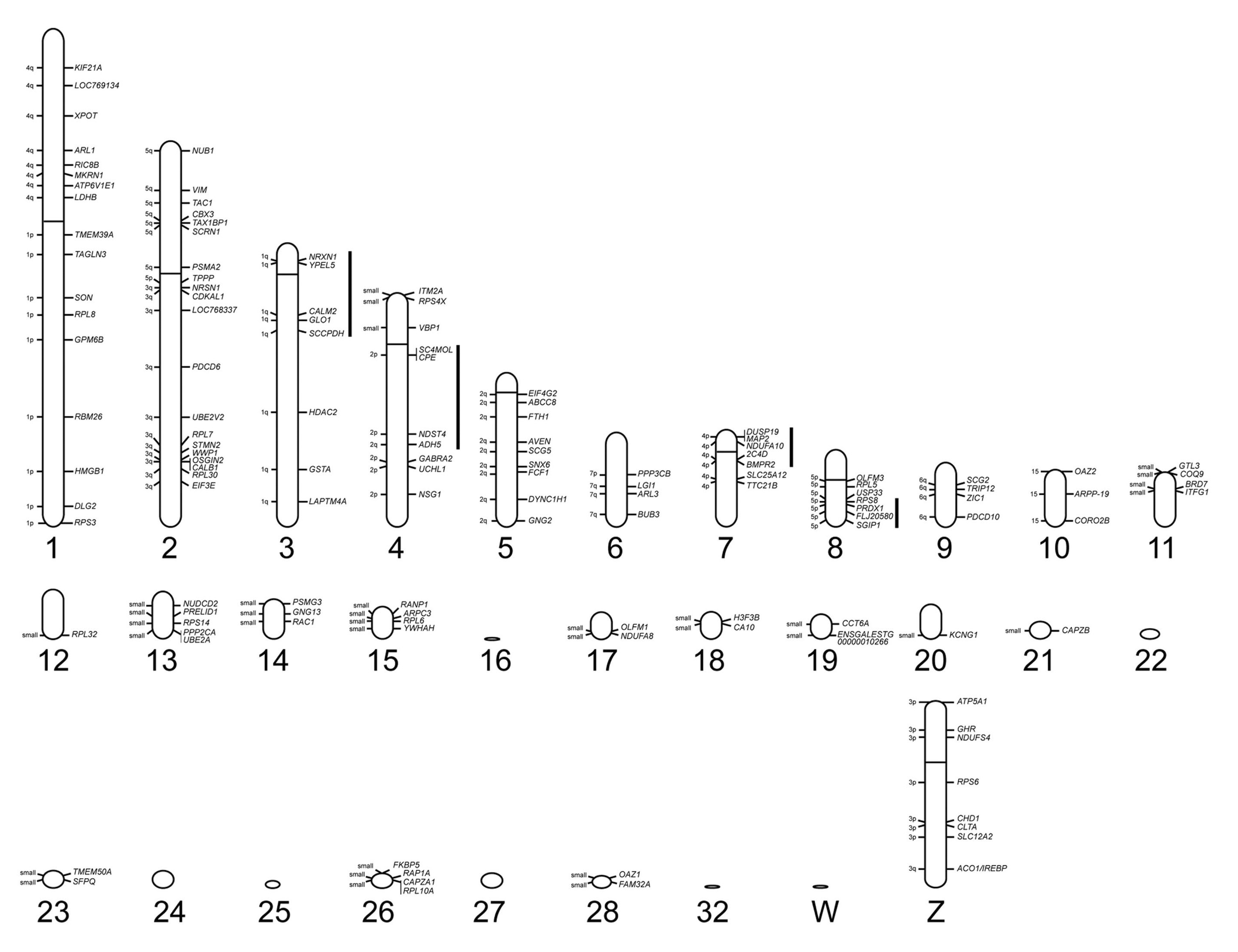

Supplement: Figure S3 — Comparative map of chicken homologs of C. siamensis genes. Chromosomal locations of chicken homologs were identified using the BLASTN programs of Ensembl and/or NCBI (retrieved in March 2012). Horizontal bars inside chromosomes represent the locations of centromeres. Solid vertical bars to the right of chromosomes indicate the chromosomal regions in which intrachromosomal rearrangements occurred between chicken and crocodile. (TIF) [file pone.0053027.s003.tif]

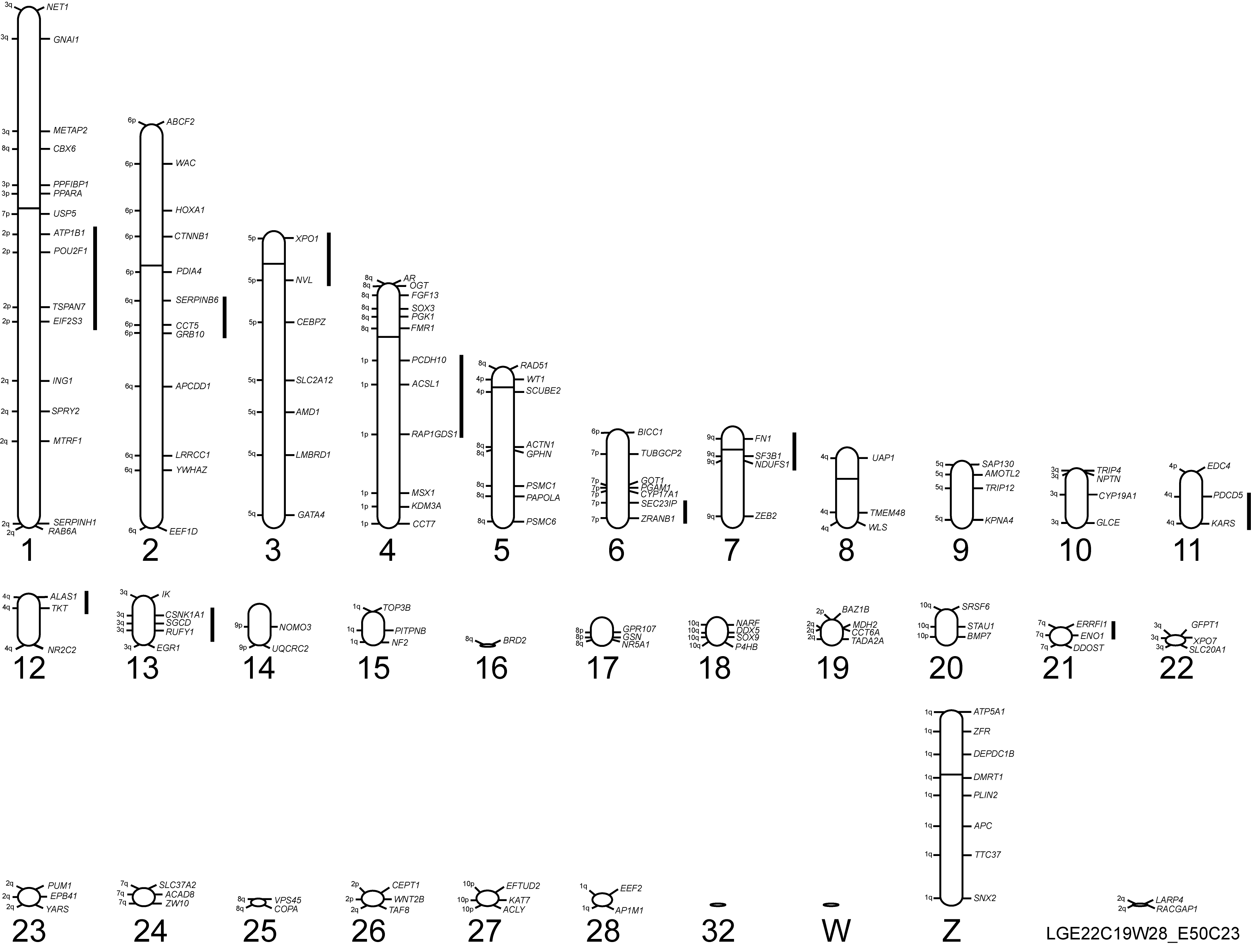

Supplement: Figure S4 — Comparative map of chicken homologs of Xenopus genes. Chromosomal locations of chicken homologs were identified using the BLASTN programs of Ensembl and/or NCBI (retrieved in March 2012). Horizontal bars inside chromosomes represent the locations of centromeres. Solid vertical bars to the right of chromosomes indicate the chromosomal regions in which intrachromosomal rearrangements occurred between chicken and X. tropicalis. (TIF) [file pone.0053027.s004.tif]

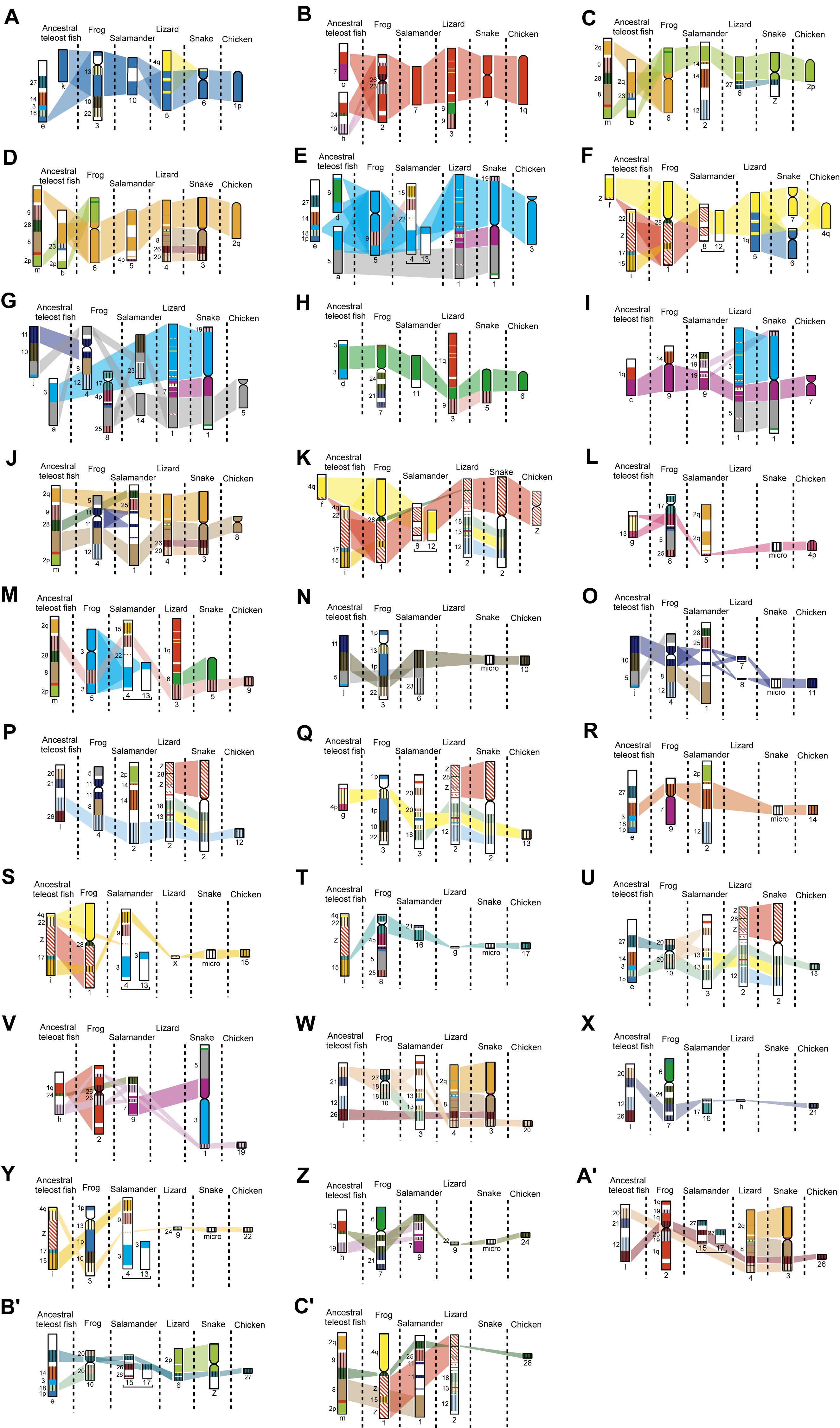

Supplement: Figure S5 — Comparison of chromosomal locations of chicken chromosomal linkages among frog, salamander, lizard, snake, and ancestral teleost fish. Comparison of chromosomal locations of chicken macrochromosomal linkages (GGA1p, 1q, 2p, 2q, 3, 4q, GGA5–8, and Z) (A–K) and microchromosomal linkages (GGA4p, 9–15, 17–22, 24, and 26–28) (L–C’) among the frog (X. tropicalis), salamander (Ambystoma mexicanum/A. tigrinum), lizard (Anolis carolinensis), snake (E. quadrivirgata), and ancestral teleost fish. Genetic linkages of chicken macro- and microchromosomes are represented by the same colored bars as those in Figure 5, and each conserved genetic linkage was defined when two or more genes were located on each of chicken chromosomes. The numbers of the homologous chicken chromosomes are shown to the left of the chromosomes. Eleven linkages of chicken macrochromosomes (1p, 1q, 2p, 2q, 3, 4q, GGA5–8, and Z) have been highly conserved in two squamates, and 10 of the 11 macrochromosomal linkages, with the exception of GGA5, were also conserved in X. tropicalis and the salamander. In contrast, the linkage homology was found to be much lower between chicken macrochromosomes and the ancestral teleost fish chromosomes. Five chicken microchromosomal linkages (GGA9, 12, 20, 26, and 27) were each integrated into chromosomes that were nonhomologous between amphibians and squamates. However, linkage homologies with respect to sites of integration were found for four linkage groups (corresponding to GGA13, 18, 19, and 28) between amphibians and squamates. The genetic linkages of GGA13 and GGA18 were co-located on single chromosomes of the salamander (chromosome 3) and the two squamates (chromosome 2). In addition, the genetic linkages of GGA19 and GGA7 were co-located on salamander chromosome 9 and snake chromosome 1, and those of GGA28 and GGAZ on Xenopus chromosome 1 and lizard chromosome 2. Nine genetic linkages (corresponding to GGA4p, 10, 11, 14, 15, 17, 21, 22, and 24) have remained as microc [file pone.0053027.s005.tif]
