# Supplementary material for: Inference of the Protokaryotypes of Amniotes and Tetrapods and the Evolutionary Processes of Microchromosomes from Comparative Gene Mapping
Source: PLoS One. 2012 Dec 31;7(12):e53027. doi: 10.1371/journal.pone.0053027 (PMC3534110; doi:10.1371/journal.pone.0053027)
Supplement: Table S1 — List of 162 genes that were localized to chromosomes of P. sinensis. (DOC) [file pone.0053027.s006.doc]

| **Table S1** |  |  |  |  |  |  |  |
| --- | --- | --- | --- | --- | --- | --- | --- |
| List of 162 genes that were localized to chromosomes of *Pelodiscus sinensis.* | | | | |  |  |  |
| Gene symbol^*^ | Insert size (kb) | Sequence length (bp) | E-value^†^ | Accession no. | Chromosomal location | | |
|  |  |  |  |  | turtle | chicken^‡^ | human^‡^ |
| *TAF3* | 1.4 | 532 | 5.8e-99 | FS943139 | 1p | 1p | 10p15.1 |
| *NAPE-PLD* | 2.2 | 779 | 7.0e-199 | FS943140 | 1p | 1p | 7q22.1 |
| *PMPCB* | 1.6 | 464 | 3.2e-128 | FS943141 | 1p | 1p | 7q22.1 |
| *NAV3*^§^ | 1.2 | 1033 | 1.4e-141 | AU312263 | 1p | 1p | 12q14.3 |
| *NAP1L1*^§^ | 2.0 | 811 | 5.1e-90 | AU312281 | 1p | 1p | 12q21.2 |
| *SYT1* | 2.0 | 519 | 2.4e-148 | FS943206 | 1p | 1p | 12cen-q21 |
| *MAP3K7IP1*^§^ | 1.0 | 561 | 1.5e-121 | AU312271 | 1p | 1p | 22q13.1 |
| *MFNG*^§^ | 0.8 | 811 | 6.6e-80 | AB188370 | 1p | 1p | 22q12 |
| *RPL3*^§^ | 1.6 | 498 | 1.7e-120 | AU312265 | 1p | 1p | 22q13 |
| *TRA1*^§^ | 2.5 | 531 | 1.2e-86 | AU312248 | 1p | 1p | 7q21.2-q22.1 |
| *MKRN1* | 1.4 | 780 | 3.4e-105 | FS943142 | 1p | 1p | 7q34 |
| *PTN*^§^ | 2.2 | 516 | 2.4e-109 | AU312250 | 1p | 1p | 7q33 |
| *KIAA0153*^§^ | 1.8 | 809 | 1.5e-78 | AU312266 | 1p | 1p | 22q13.31 |
| *CCND2* | 3.0 | 520 | 6.7e-121 | FS943208 | 1p | 1p | 12p13 |
| *USP5*^§^ | 1.2 | 800 | 7.8e-84 | AU312276 | 1q | 1q | 12p13 |
| *DPT*^§^ | 1.2 | 680 | 3.3e-77 | AU312278 | 1q | 1q | 1q12-q23 |
| *ZNF294*^§^ | 2.0 | 543 | 3.0e-29 | AU312299 | 1q | 1q | 21q22.11 |
| *RPL8*^§^ | 1.0 | 824 | 1.9e-98 | AU312288 | 1q | 1q | 8q24.3 |
| *C21orf33*^§^ | 2.5 | 655 | 1.2e-102 | AU312295 | 1q | 1q | 21q22.3 |
| *TSPAN7* | 2.0 | 724 | 3.7e-103 | FS943143 | 1q | 1q | Xp11.4 |
| *PDHA1* | 1.5 | 631 | 5.3e-37 | FS943144 | 1q | 1q | Xp22.1 |
| *EIF2S3*^§^ | 2.0 | 658 | 4.3e-109 | AU312268 | 1q | 1q | Xp22.2-p22.1 |
| *MAB21L1* | 1.8 | 784 | 0.0e+00 | FS943145 | 1q | 1q | 13q13 |
| *WBP4* | 3.2 | 739 | 4.0e-22 | FS943146 | 1q | 1q | 13q14.11 |
| *WNT11*^§^ | 1.2 | 1227 | 8.4e-146 | AB188366 | 1q | 1q | 11q13.5 |
| *RAB6A* | 2.3 | 777 | 2.8e-84 | FS943147 | 1q | 1q | 11q13.3 |
| *ARF1*^§^ | 2.0 | 649 | 5.7e-87 | AU312289 | 2p | 2p | 1q42 |
| *GARS*^§^ | 2.0 | 802 | 2.3e-104 | AU312286 | 2p | 2p | 7p15 |
| *DNAJB6* | 2.4 | 567 | 2.7e-73 | FS943148 | 2p | 2p | 7q36.3 |
| *DFNA5* | 1.8 | 756 | 3.4e-140 | FS943149 | 2p | 2p | 7p15 |
| *RSU1* | 0.9 | 736 | 2.2e-72 | FS943150 | 2p | 2p | 10p13 |
| *RARB*^§^ | 1.5 | 1461 | 1.0e-146 | AB188354 | 2p | 2p | 3p24 |
| *SHH*^§^ | 0.4 | 415 | 6.1e-173 | AB181135 | 2p | 2p | 7q36 |
| *LAMR1*^§^ | 1.0 | 829 | 2.4e-164 | AU312259 | 2p | 2p | 3p22.2 |
| *CCT5* | 1.4 | 658 | 2.5e-80 | FS943151 | 2p | 2q | 5p15.2 |
| *NEDD9* | 4.0 | 777 | 2.0e-138 | FS943152 | 2q | 2q | 6p25-p24 |
| *NRN1* | 1.6 | 792 | 4.9e-104 | FS943153 | 2q | 2q | 6p25.1 |
| *MBP* | 3.0 | 531 | 4.3e-70 | FS943205 | 2q | 2q | 18q23 |
| *RPL7* | 0.9 | 525 | 3.1e-91 | FS943154 | 2q | 2q | 8q21.11 |
| *APCDD1*^§^ | 2.2 | 2151 | 2.1e-194 | AB124565 | 2q | 2q | 18p11.22 |
| *COLEC12* | 1.7 | 759 | 6.7e-44 | FS943155 | 2q | 2q | 18pter-p11.3 |
| *TTR* | 2.1 | 514 | 8.5e-20 | FS943207 | 2q | 2q | 18q12.1 |
| *NSMAF*^§^ | 4.0 | 521 | 4.6e-86 | AU312241 | 2q | 2q | 8q12-q13 |
| *RUNX1T1* | 1.4 | 601 | 5.6e-224 | FS943156 | 2q | 2q | 8q22 |
| *EIF3S6*^§^ | 1.6 | 548 | 3.4e-81 | AU312274 | 2q | 2q | 8q22-q23 |
| *LRPPRC* | 2.2 | 781 | 1.0e-55 | FS943157 | 3p | 3q | 2p21 |
| *BMP2*^§^ | 0.7 | 656 | 5.0e-251 | AB181137 | 3p | 3q | 20p12 |
| *ACTR2* | 2.1 | 800 | 5.8e-141 | FS943158 | 3p | 3p | 2p14 |
| *RTN4* | 1.5 | 635 | 3.1e-148 | FS943159 | 3p | 3p | 2p16.3 |
| *XPO1*^§^ | 2.3 | 573 | 2.2e-111 | AU312293 | 3p | 3p | 2p16 |
| *EPHX1*^§^ | 1.7 | 737 | 4.8e-120 | AU312282 | 3p | 3q | 1q42.1 |
| *NVL*^§^ | 2.0 | 794 | 1.0e-112 | AU312294 | 3p | 3q | 1q41-q42.2 |
| *TMEM63A* | 3.2 | 774 | 3.0e-107 | FS943160 | 3p | 3q | 1q42.12 |
| *TARBP1* | 2.5 | 674 | 3.1e-41 | FS943161 | 3q | 3q | 1q42.3 |
| *EEF1A1* | 1.8 | 792 | 4.7e-213 | FS943162 | 3q | 3q | 6q14.1 |
| *LMBRD1* | 2.4 | 711 | 7.7e-58 | FS943163 | 3q | 3q | 6q13 |
| *RPS7* | 0.8 | 630 | 6.2e-113 | FS943164 | 3q | 3q | 2p25 |
| *RNASEH1*^§^ | 3.0 | 520 | 3.4e-30 | AU312243 | 3q | 3q | 2p25 |
| *ALB* | 2.1 | 635 | 4.2e-38 | FS943165 | 4p | 4q | 4q13.3 |
| *PAPSS1*^§^ | 1.5 | 810 | 2.3e-144 | AU312290 | 4q | 4q | 4q24 |
| *LEF1*^§^ | 2.4 | 2381 | 7.3e-123 | AB124566 | 4q | 4q | 4q23-q25 |
| *ANK2* | 3.0 | 1473 | 4.5e-160 | FS943203 | 4q | 4q | 4q25-q27 |
| *PCDH18* | 2.1 | 707 | 3.6e-263 | FS943166 | 4q | 4q | 4q31 |
| *GUCY1B3* | 3.0 | 528 | 2.3e-145 | FS943204 | 4q | 4q | 4q31.3-q33 |
| *RPS3A* | 1.0 | 703 | 2.9e-150 | FS943167 | 4q | 4q | 4q31.2-q31.3 |
| *C4orf27* | 1.4 | 656 | 9.5e-100 | FS943168 | 4q | 4q | 4q33 |
| *HMGB2*^§^ | 1.8 | 901 | 4.5e-114 | AU312262 | 4q | 4q | 4q31 |
| *FAT*^§^ | 2.1 | 527 | 7.6e-87 | AU312273 | 4q | 4q | 4q35 |
| *UCHL1*^§^ | 3.0 | 532 | 3.2e-33 | AU312247 | 4q | 4q | 4p14 |
| *RPL9* | 1.3 | 636 | 5.4e-67 | FS943169 | 4q | 4q | 4p13 |
| *MSX1*^§^ | 1.4 | 1448 | 0.0e+00 | AB124572 | 4q | 4q | 4p16.3-p16.1 |
| *MYOD1*^§^ | 1.2 | 1227 | 4.8e-196 | AB188356 | 5p | 5q | 11p15.4 |
| *SBF2* | 1.9 | 748 | 1.5e-87 | FS943170 | 5p | 5q | 11p15.4 |
| *CAT* | 2.3 | 552 | 1.0e-113 | FS943171 | 5q | 5q | 11p13 |
| *EIF2S1*^§^ | 1.5 | 461 | 7.9e-166 | AU312298 | 5q | 5q | 14q23.3 |
| *ACTC1*^§^ | 1.5 | 739 | 1.5e-237 | AU312292 | 5q | 5q | 15q11-q14 |
| *COQ6*^§^ | 1.0 | 925 | 1.4e-18 | AU312260 | 5q | 5q | 14q24.3 |
| *PAX9*^§^ | 0.5 | 538 | 2.2e-77 | AB181136 | 5q | 5q | 14q13.3 |
| *EIF2B2*^§^ | 2.1 | 839 | 1.5e-70 | AU312287 | 5q | 5q | 14q24.3 |
| *PRRX1*^§^ | 0.5 | 541 | 2.9e-103 | AB188347 | 5q | 8p | 1q24 |
| *CDCA4* | 1.8 | 810 | 1.0e-295 | FS943172 | 5q | 5q | 14q32.33 |
| *CKB* | 1.5 | 766 | 1.1e-109 | FS943173 | 5q | 5q | 14q32 |
| *BMP4*^§^ | 0.9 | 907 | 7.7e-173 | AB181138 | 5q | 5q | 14q22-q23 |
| *C14orf166*^§^ | 1.0 | 687 | 3.3e-74 | AU312301 | 5q | 5q | 14q22.1 |
| *CLTA*^§^ | 1.0 | 799 | 9.8e-166 | AU312285 | 6p | Zq | 9p13 |
| *C9orf82* | 2.0 | 668 | 6.0e-119 | FS943174 | 6q | Zq | 9p21.2 |
| *ACO1/IREBP*^§^ | 1.1 | 1122 | 2.4e-95 | AB185397 | 6q | Zq | 9p21.1 |
| *MTAP* | 2.0 | 586 | 4.6e-138 | FS943175 | 6q | Zq | 9p21 |
| *RPS6*^§^ | 0.6 | 593 | 6.4e-147 | AB266736 | 6q | Zq | 9p21 |
| *DMRT1*^§^ | 1.2 | 1164 | 1.4e-206 | AB179697 | 6q | Zq | 9p24.3 |
| *ALDH7A1*^§^ | 1.7 | 616 | 1.2e-54 | AU312269 | 6q | Zq | 5q31 |
| *FBP1*^§^ | 1.5 | 732 | 2.8e-63 | AU312291 | 6q | Zq | 9q22.3 |
| *SNX2* | 2.0 | 791 | 2.0e-89 | FS943176 | 6q | Zq | 5q23 |
| *CHD1*^§^ | 2.0 | 798 | 4.5e-123 | AU312270 | 6q | Zq | 5q15-q21 |
| *RGMB* | 1.7 | 608 | 3.5e-296 | FS943177 | 6q | Zq | 5q15 |
| *GHR*^§^ | 1.3 | 1310 | 2.5e-250 | AB267378, AB267379 | 6q | Zp | 5p13-p12 |
| *SIAT8C*^§^ | 1.4 | 420 | 2.3e-262 | AU312252 | 6q | Zp | 18q21.31 |
| *FGF10*^§^ | 1.5 | 1465 | 1.5e-167 | AB124573 | 6q | Zp | 5p13-p12 |
| *ATP5A1*^§^ | 1.0 | 990 | 2.5e-177 | AB266737, AB266738 | 6q | Zp | 18q12-q21 |
| *NARS* | 2.0 | 723 | 4.1e-102 | FS943178 | 6q | Zp | 18q21.31 |
| *NDUFS1* | 2.0 | 570 | 6.3e-125 | FS943179 | 7p | 7q | 2q33-q34 |
| *EEF1B2* | 0.9 | 680 | 3.0e-84 | FS943180 | 7p | 7q | 2q33.3 |
| *FN1* | 1.2 | 721 | 2.5e-77 | FS943181 | 7p | 7p | 2q34 |
| *HOXD13*^§^ | 1.1 | 1144 | 7.2e-217 | AB188346 | 7q | 7q | 2q31.1 |
| *SP5*^§^ | 2.1 | 2079 | 3.1e-300 | AB124563 | 7q | 7q | 2q31.1 |
| *ZFHX1B* | 3.0 | 800 | 0.0e+00 | FS943182 | 7q | 7q | 2q22 |
| *CCNT2* | 2.4 | 703 | 8.8e-126 | FS943183 | 7q | 7q | 2q21.3 |
| *EN1*^§^ | 0.6 | 639 | 2.4e-212 | AB188348 | 7q | 7q | 2q14.2 |
| *ADCY5* | 2.8 | 780 | 5.4e-174 | FS943184 | 7q | 7q | 3q13.2-q21 |
| *GLI2*^§^ | 1.1 | 1126 | 1.4e-241 | AB188357 | 7q | 7q | 2q14 |
| *CDC2* | 1.4 | 632 | 5.9e-101 | FS943185 | 8q | 6 | 10q21.1 |
| *EMX2*^§^ | 1.0 | 985 | 1.5e-132 | AB188349 | 8q | 6 | 10q26.1 |
| *C10orf46* | 4.3 | 605 | 4.2e-100 | FS943186 | 8q | 6 | 10q26.11 |
| *FGFR2*^§^ | 1.4 | 1432 | 3.2e-122 | AB188372 | 8q | 6 | 10q26 |
| *COX15*^§^ | 1.2 | 519 | 1.6e-34 | AU312249 | 8q | 6 | 10q24 |
| *FGF8*^§^ | 1.5 | 1495 | 7.4e-221 | AB124574 | 8q | 6 | 10q24 |
| *NF2*^§^ | 1.6 | 1562 | 2.2e-92 | AB478250 | Z | 15 | 22q12.2 |
| *SF3A1*^§^ | 0.9 | 917 | 7.1e-102 | AB478252 | Z | 15 | 22q12.2 |
| *TOP3B*^§^ | 1.3 | 1323 | 1.4e-86 | AU312272 | Z | 15 | 22q11.22 |
| *SBNO1*^§^ | 0.7 | 651 | 3.0e-115 | AB478253 | Z | 15 | 12q24.31 |
| *GIT2*^§^ | 1.4 | 1387 | 1.1e-110 | AB478251 | Z | 15 | 12q24.1 |
| *COL4A5*^§^ | 2.2 | 795 | 1.8e-104 | AU312283 | micro | 4p | Xq22 |
| *DCX*^§^ | 1.7 | 1125 | 3.0e-238 | AU312264 | micro | 4p | Xq22.3-q23 |
| *PGK1* | 1.3 | 808 | 6.0e-121 | FS943187 | micro | 4p | Xq13 |
| *FGF13* | 2.3 | 585 | 4.7e-80 | FS943188 | micro | 4p | Xq27 |
| *CETN2* | 1.4 | 470 | 6.5e-88 | FS943189 | micro | 4p | Xq28 |
| *CACYBP* | 1.5 | 723 | 1.1e-96 | FS943190 | micro | 8p | 1q24-q25 |
| *SCG2*^§^ | 2.3 | 636 | 1.4e-208 | AU312275 | micro | 9 | 2q35-q36 |
| *RASA2*^§^ | 3.0 | 561 | 1.1e-101 | AU312254 | micro | 9 | 3q22-q23 |
| *PLD1*^§^ | 3.0 | 517 | 4.1e-27 | AU312251 | micro | 9 | 3q26 |
| *RNF13* | 2.3 | 754 | 5.0e-67 | FS943191 | micro | 9 | 3q25.1 |
| *DBR1* | 1.8 | 635 | 1.0e-90 | FS943192 | micro | 9 | 3q22.3 |
| *PAX3*^§^ | 2.0 | 1957 | 1.1e-171 | AB188350 | micro | 9 | 2q35 |
| *CRABP1*^§^ | 0.8 | 752 | 6.8e-125 | AB124564 | micro | 10 | 15q24 |
| *KARS*^§^ | 1.5 | 516 | 1.3e-106 | AU312242 | micro | 11 | 16q23-q24 |
| *RBM5* | 1.1 | 790 | 5.8e-89 | FS943193 | micro | 12 | 3p21.3 |
| *TKT* | 1.8 | 516 | 5.6e-124 | FS943194 | micro | 12 | 3p14.3 |
| *WNT7A*^§^ | 1.0 | 1038 | 0.0e+00 | AB188364 | micro | 12 | 3p25 |
| *WNT5A*^§^ | 1.5 | 1521 | 0.0e+00 | AB188363 | micro | 12 | 3p21-p14 |
| *CTNNA1*^§^ | 4.5 | 520 | 1.1e-160 | AU312240 | micro | 13 | 5q31 |
| *SKP1A*^§^ | 1.8 | 815 | 9.6e-144 | AU312280 | micro | 13 | 5q31 |
| *SPARC*^§^ | 2.0 | 513 | 5.8e-79 | AU312255 | micro | 13 | 5q31.3-q32 |
| *CSNK1A1*^§^ | 2.0 | 449 | 5.4e-101 | AU312296 | micro | 13 | 5q32 |
| *DDX46*^§^ | 1.1 | 1052 | 5.8e-119 | AB188384 | micro | 13 | 5q31.1 |
| *UQCRC2* | 1.5 | 795 | 8.7e-42 | FS943195 | micro | 14 | 16p12 |
| *LFNG*^§^ | 1.0 | 998 | 2.8e-113 | AB188368 | micro | 14 | 7p22.2 |
| *LHX2*^§^ | 1.5 | 710 | 3.3e-296 | AU312297 | micro | 17 | 9q33.3 |
| *GSN* | 1.2 | 658 | 4.8e-59 | FS943196 | micro | 17 | 9q33 |
| *RPL7A* | 1.0 | 716 | 8.3e-100 | FS943197 | micro | 17 | 9q34 |
| *DDX5* | 2.5 | 792 | 2.0e-118 | FS943198 | micro | 18 | 17q21 |
| *PMP22* | 1.9 | 550 | 1.4e-77 | FS943199 | micro | 18 | 17p12 |
| *BAZ1B*^§^ | 2.0 | 756 | 5.9e-155 | AU312277 | micro | 19 | 7q11.23 |
| *GTF2I*^§^ | 2.0 | 761 | 7.2e-90 | AU312279 | micro | 19 | 7q11.23 |
| *TADA2A* | 1.2 | 784 | 1.3e-61 | FS943200 | micro | 19 | 17q12-q21 |
| *BMP7*^§^ | 0.8 | 772 | 6.1e-107 | AB188367 | micro | 20 | 20q13 |
| *SKI*^§^ | 1.2 | 1232 | 1.6e-126 | AB188380 | micro | 21 | 1q22-q24 |
| *PAX7*^§^ | 1.8 | 1786 | 5.0e-184 | AB188351 | micro | 21 | 1p36.13 |
| *SLC20A1*^§^ | 3.0 | 740 | 9.3e-37 | AU312245 | micro | 22 | 2q11-q14 |
| *STMN1* | 1.0 | 785 | 1.7e-141 | FS943201 | micro | 23 | 1p36.11 |
| *WNT2B*^§^ | 1.8 | 1795 | 1.6e-198 | AB188360 | micro | 26 | 1p13 |
| *EEF2*^§^ | 1.2 | 526 | 1.2e-118 | AU312258 | micro | 28 | 19pter-q12 |
| *CIRBP* | 1.4 | 745 | 2.9e-143 | FS943202 | micro | un | 19p13.3 |
| ^*^Human gene symbol. ^†^E-values of reptile homologues versus chicken genes obtained with the BLAST and/or TBLASTX program of Ensembl (retrieved in March 2012).  ^‡^Chromosomal locations of chicken and human homologues obtained with the BLASTN programs of Ensembl and/or NCBI (retrieved in March 2012). un, unknown chromosomal location. no, no homologues were found.  ^§^Genes mapped in our previous studies [1–4].  References  1. Matsuda Y, Nishida-Umehara C, Tarui H, Kuroiwa A, Yamada K, et al. (2005) Highly conserved linkage homology between birds and turtles: Bird and turtle chromosomes are precise counterparts of each other. Chromosome Res 13: 601–615.  2. Kuraku S, Ishijima J, Nishida-Umehara C, Agata K, Kuratani S, et al. (2006) cDNA-based gene mapping and GC3 profiling in the soft-shelled turtle suggest a chromosomal size-dependent GC bias shared by sauropsids. Chromosome Res 14: 187–202.  3. Kawai A, Nishida-Umehara C, Ishijima J, Tsuda Y, Ota H, et al. (2007) Different origins of bird and reptile sex chromosomes inferred from comparative mapping of chicken Z-linked genes. Cytogenet Genome Res 117: 92–102.  4. Kawagoshi T, Uno Y, Matsubara K, Matsuda Y, Nishida C (2009) The ZW micro-sex chromosomes of the Chinese soft-shelled turtle (*Pelodiscus sinensis*, Trionychidae, Testudines) have the same origin as chicken chromosome 15. Cytogenet Genome Res 125: 125–131. | | | | | | | |
