# Supplementary material for: Inference of the Protokaryotypes of Amniotes and Tetrapods and the Evolutionary Processes of Microchromosomes from Comparative Gene Mapping
Source: PLoS One. 2012 Dec 31;7(12):e53027. doi: 10.1371/journal.pone.0053027 (PMC3534110; doi:10.1371/journal.pone.0053027)
Supplement: Table S2 — List of 131 genes that were localized to chromosomes of C. siamensis. (DOC) [file pone.0053027.s007.doc]

| **Table S2** |  |  |  |  |  |  |  |
| --- | --- | --- | --- | --- | --- | --- | --- |
| List of 131 genes that were localized to chromosomes of *Crocodylus siamensis.* | | | | |  |  |  |
| Gene symbol^*^ | Insert size (kb) | Sequence length (bp) | E-value^†^ | Accession no. | Chromosomal　location | | |
|  |  |  |  |  | crocodile | chicken^‡^ | human^‡^ |
| *RPS3* | 0.7 | 523 | 3.9e-131 | FS943209 | 1p | 1q | 11q13.3-q13.5 |
| *DLG2* | 1.1 | 691 | 3.7e-74 | FS943210 | 1p | 1q | 11q14.1 |
| *HMGB1* | 1.5 | 664 | 7.0e-136 | FS943211 | 1p | 1q | 13q12 |
| *RBM26* | 1.0 | 752 | 1.1e-138 | FS943212 | 1p | 1q | 13q31.1 |
| *GPM6B* | 1.9 | 674 | 9.1e-121 | FS943213 | 1p | 1q | Xp22.2 |
| *RPL8* | 1.0 | 692 | 3.4e-157 | FS943214 | 1p | 1q | 8q24.3 |
| *SON* | 1.4 | 674 | 6.1e-279 | FS943215 | 1p | 1q | 21q22.11 |
| *TAGLN3* | 1.1 | 665 | 5.6e-124 | FS943216 | 1p | 1q | 3q13.2 |
| *TMEM39A* | 0.7 | 467 | 6.8e-169 | FS943217 | 1p | 1q | 3q13.33 |
| *CALM2* | 1.3 | 741 | 4.7e-194 | FS943218 | 1q | 3q | 2p21 |
| *NRXN1* | 1.1 | 707 | 7.6e-298 | FS943219 | 1q | 3p | 2p16.3 |
| *YPEL5* | 0.6 | 425 | 7.7e-172 | FS943220 | 1q | 3p | 2p23.1 |
| *SCCPDH* | 1.2 | 730 | 4.6e-41 | FS943221 | 1q | 3q | 1q44 |
| *GLO1* | 0.9 | 571 | 6.7e-61 | FS943222 | 1q | 3q | 6p21.3-p21.1 |
| *HDAC2* | 2.0 | 533 | 4.5e-85 | FS943223 | 1q | 3q | 6q21 |
| *GSTA* | 1.1 | 707 | 7.0e-84 | FS943224 | 1q | 3q | 1q21 |
| *LAPTM4A* | 1.5 | 683 | 2.3e-64 | FS943225 | 1q | 3q | 2p24.1 |
| *NSG1* | 0.9 | 732 | 4.6e-259 | FS943226 | 2p | 4q | 4p16.3 |
| *GABRA2* | 1.3 | 610 | 9.7e-95 | FS943227 | 2p | 4q | 4p12 |
| *UCHL1* | 1.0 | 777 | 3.3e-60 | FS943228 | 2p | 4q | 4p14 |
| *CPE* | 0.9 | 550 | 3.3e-117 | FS943229 | 2p | 4q | 4q32.3 |
| *SC4MOL* | 1.6 | 661 | 6.2e-109 | FS943230 | 2p | 4q | 4q32-q34 |
| *NDST4* | 1.1 | 663 | 4.8e-101 | FS943231 | 2p | 4q | 4q26 |
| *RPL34* | 0.6 | 369 | 3.3e-59 | FS943232 | 2p | un | 4q25 |
| *ADH5* | 1.2 | 739 | 2.5e-88 | FS943233 | 2q | 4q | 4q23 |
| *FTH1* | 0.9 | 654 | 3.6e-92 | FS943234 | 2q | 5q | 11q13 |
| *EIF4G2* | 1.2 | 719 | 1.8e-154 | FS943235 | 2q | 5q | 11p15 |
| *ABCC8* | 1.2 | 683 | 5.1e-80 | FS943236 | 2q | 5q | 11p15.1 |
| *FCF1* | 0.9 | 683 | 1.3e-27 | FS943237 | 2q | 5q | 14q24.3 |
| *AVEN* | 1.4 | 758 | 6.0e-37 | FS943238 | 2q | 5q | 15q13.1 |
| *SCG5* | 1.4 | 705 | 5.2e-86 | FS943239 | 2q | 5q | 15q13-q14 |
| *SNX6* | 1.1 | 605 | 1.2e-113 | FS943240 | 2q | 5q | 14q13.1 |
| *DYNC1H1* | 1.1 | 677 | 3.2e-108 | FS943241 | 2q | 5q | 14q32 |
| *GNG2* | 1.0 | 629 | 1.4e-102 | FS943242 | 2q | 5q | 14q21 |
| *ATP5A1*^¶^ | 1.0 | 990 | 1.3e-102 | AB266730, AB266726 | 3p | Zp | 18q12-q21 |
| *NDUFS4* | 0.8 | 574 | 3.2e-117 | FS943243 | 3p | Zp | 5q11.1 |
| *GHR*^¶^ | 1.3 | 1310 | 2.5e-202 | AB266728, AB266727 | 3p | Zp | 5p13-p12 |
| *CLTA* | 1.0 | 591 | 1.8e-157 | FS943244 | 3p | Zq | 9p13 |
| *CHD1*^¶^ | 1.3 | 1260 | 6.5e-143 | AB266725, AB266724 | 3p | Zq | 5q15-q21 |
| *SLC12A2* | 0.7 | 486 | 7.5e-43 | FS943245 | 3p | Zq | 5q23.3 |
| *RPS6* | 0.9 | 710 | 2.1e-121 | FS943246 | 3p | Zq | 9p21 |
| *ACO1/IREBP*^¶^ | 1.1 | 1122 | 3.0e-119 | AB266722, AB266723 | 3q | Zq | 9p21.1 |
| *PDCD6* | 1.0 | 720 | 2.1e-70 | FS943247 | 3q | 2q | 5p15.33 |
| *CDKAL1* | 1.4 | 703 | 4.1e-123 | FS943248 | 3q | 2q | 6p22.3 |
| *NRSN1* | 1.6 | 700 | 6.2e-246 | FS943249 | 3q | 2q | 6p22.3 |
| *LOC768337*^§^ | 2.2 | 665 | 0.0e+00 | FS943250 | 3q | 2q | no |
| *UBE2V2* | 1.2 | 698 | 0.0e+00 | FS943251 | 3q | 2q | 8q11.21 |
| *STMN2* | 0.7 | 517 | 6.1e-141 | FS943252 | 3q | 2q | 8q21.13 |
| *OSGIN2* | 0.9 | 657 | 5.8e-235 | FS943253 | 3q | 2q | 8q21 |
| *RPL7* | 0.9 | 632 | 2.5e-86 | FS943254 | 3q | 2q | 8q21.11 |
| *RPL30* | 0.6 | 412 | 5.4e-101 | FS943255 | 3q | 2q | 8q22 |
| *WWP1* | 1.6 | 708 | 5.1e-87 | FS943256 | 3q | 2q | 8q21 |
| *CALB1* | 1.2 | 702 | 1.5e-52 | FS943257 | 3q | 2q | 8q21.3-q22.1 |
| *EIF3E* | 2.0 | 641 | 2.0e-87 | FS943258 | 3q | 2q | 8q22-q23 |
| *TTC21B* | 1.4 | 626 | 8.3e-119 | FS943259 | 4p | 7q | 2q24.3 |
| *SLC25A12* | 1.4 | 674 | 5.0e-112 | FS943260 | 4p | 7q | 2q24 |
| *2C4D* | 1.2 | 621 | 6.7e-110 | FS943261 | 4p | 7q | 2q33.1 |
| *DUSP19* | 1.9 | 721 | 3.7e-112 | FS943262 | 4p | 7p | 2q32.1 |
| *BMPR2* | 0.8 | 638 | 1.2e-195 | FS943263 | 4p | 7q | 2q33-q34 |
| *MAP2* | 0.9 | 627 | 7.7e-153 | FS943264 | 4p | 7p | 2q34-q35 |
| *NDUFA10* | 1.4 | 688 | 4.1e-58 | FS943265 | 4p | 7p | 2q37.3 |
| *LDHB* | 1.0 | 653 | 2.3e-113 | FS943266 | 4q | 1p | 12p12.2-p12.1 |
| *ATP6V1E1* | 1.5 | 732 | 1.7e-76 | FS943267 | 4q | 1p | 22q11.1 |
| *RIC8B* | 1.1 | 705 | 5.3e-78 | FS943268 | 4q | 1p | 12q23.3 |
| *MKRN1* | 0.9 | 689 | 5.3e-171 | FS943269 | 4q | 1p | 7q34 |
| *ARL1* | 1.0 | 714 | 6.2e-69 | FS943270 | 4q | 1p | 12q23.2 |
| *XPOT* | 1.3 | 721 | 1.2e-102 | FS943271 | 4q | 1p | 12q14.2 |
| *LOC769134*^§^ | 0.9 | 665 | 6.0e-69 | FS943272 | 4q | 1p | no |
| *KIF21A* | 1.8 | 711 | 4.3e-110 | FS943273 | 4q | 1p | 12q12 |
| *RPS8* | 0.7 | 507 | 1.5e-125 | FS943274 | 5p | 8q | 1p34.1-p32 |
| *PRDX1* | 1.1 | 704 | 1.6e-97 | FS943275 | 5p | 8q | 1p34.1 |
| *SGIP1* | 1.2 | 690 | 3.4e-121 | FS943276 | 5p | 8q | 1p31.3 |
| *FLJ20580* | 1.1 | 638 | 6.3e-55 | FS943277 | 5p | 8q | 1p32.3 |
| *USP33* | 1.6 | 680 | 3.8e-115 | FS943278 | 5p | 8q | 1p31.1 |
| *RPL5* | 1.0 | 698 | 1.1e-144 | FS943279 | 5p | 8q | 1p22.1 |
| *OLFM3* | 1.3 | 611 | 1.5e-188 | FS943280 | 5p | 8q | 1p22 |
| *TPPP* | 1.0 | 514 | 3.5e-137 | FS943281 | 5p | 2p | 5p15.3 |
| *PSMA2* | 1.2 | 648 | 3.3e-76 | FS943282 | 5q | 2p | 7p13 |
| *TAX1BP1* | 3.1 | 689 | 3.6e-99 | FS943283 | 5q | 2p | 7p15 |
| *CBX3* | 1.2 | 668 | 3.9e-115 | FS943284 | 5q | 2p | 7p15.2 |
| *SCRN1* | 1.3 | 665 | 1.3e-91 | FS943285 | 5q | 2p | 7p14.3-p14.1 |
| *TAC1* | 1.0 | 677 | 5.1e-95 | FS943286 | 5q | 2p | 7q21-q22 |
| *VIM* | 1.2 | 696 | 2.3e-160 | FS943287 | 5q | 2p | 10p13 |
| *NUB1* | 1.5 | 736 | 1.3e-42 | FS943288 | 5q | 2p | 7q36 |
| *SCG2* | 1.4 | 617 | 6.6e-250 | FS943289 | 6q | 9 | 2q35-q36 |
| *ZIC1* | 0.9 | 658 | 3.4e-202 | FS943290 | 6q | 9 | 3q24 |
| *TRIP12* | 3.7 | 663 | 2.3e-226 | FS943291 | 6q | 9 | 2q36.3 |
| *PDCD10* | 1.3 | 651 | 1.9e-86 | FS943292 | 6q | 9 | 3q26.1 |
| *PPP3CB* | 1.5 | 650 | 3.5e-141 | FS943293 | 7p | 6 | 10q22.2 |
| *LGI1* | 1.2 | 656 | 0.0e+00 | FS943294 | 7q | 6 | 10q24 |
| *ARL3* | 0.8 | 514 | 8.7e-134 | FS943295 | 7q | 6 | 10q23.3 |
| *BUB3* | 1.1 | 726 | 3.2e-148 | FS943296 | 7q | 6 | 10q26 |
| *OAZ2* | 1.0 | 630 | 2.0e-191 | FS943297 | 15 | 10 | 15q22.31 |
| *ARPP-19* | 1.2 | 772 | 2.9e-171 | FS943298 | 15 | 10 | 15q21.2 |
| *CORO2B* | 1.0 | 701 | 3.7e-122 | FS943299 | 15 | 10 | 15q23 |
| *ITM2A* | 1.2 | 746 | 2.7e-115 | FS943300 | small | 4p | Xq13.3-Xq21.2 |
| *RPS4X* | 1.0 | 758 | 7.4e-129 | FS943301 | small | 4p | Xq13.1 |
| *VBP1* | 1.7 | 640 | 2.4e-95 | FS943302 | small | 4p | Xq28 |
| *GTL3* | 1.0 | 589 | 8.7e-85 | FS943303 | small | 11 | 16q21 |
| *COQ9* | 1.1 | 724 | 3.1e-91 | FS943304 | small | 11 | 16q21 |
| *BRD7* | 1.9 | 599 | 4.7e-64 | FS943305 | small | 11 | 16q12 |
| *ITFG1* | 2.0 | 711 | 8.2e-93 | FS943306 | small | 11 | 16q12.1 |
| *RPL32* | 1.9 | 611 | 4.9e-129 | FS943307 | small | 12 | 3p25-p24 |
| *PRELID1* | 0.6 | 430 | 5.9e-29 | FS943308 | small | 13 | 5q35.3 |
| *NUDCD2* | 1.1 | 674 | 1.7e-91 | FS943309 | small | 13 | 5q34 |
| *RPS14* | 1.1 | 698 | 1.0e-111 | FS943310 | small | 13 | 5q31-q33 |
| *UBE2B* | 1.0 | 700 | 1.4e-102 | FS943311 | small | 13 | 5q31.1 |
| *PPP2CA* | 1.6 | 535 | 4.4e-129 | FS943312 | small | 13 | 5q31.1 |
| *RAC1* | 2.1 | 688 | 7.5e-122 | FS943313 | small | 14 | 7p22 |
| *GNG13* | 1.1 | 691 | 1.8e-53 | FS943314 | small | 14 | 16p13.3 |
| *PSMG3* | 1.0 | 674 | 4.5e-99 | FS943315 | small | 14 | 7p22.3 |
| *RANP1* | 1.0 | 604 | 4.0e-141 | FS943316 | small | 15 | 6p21.33 |
| *ARPC3* | 1.1 | 628 | 1.2e-72 | FS943317 | small | 15 | 12q24.11 |
| *RPL6* | 0.9 | 592 | 1.2e-83 | FS943318 | small | 15 | 12q24.1 |
| *YWHAH* | 1.3 | 738 | 2.9e-281 | FS943319 | small | 15 | 22q12.3 |
| *NDUFA8* | 0.6 | 443 | 6.4e-88 | FS943320 | small | 17 | 9q33.2 |
| *OLFM1* | 1.4 | 543 | 3.0e-234 | FS943321 | small | 17 | 9q34.3 |
| *H3F3B* | 1.1 | 664 | 0.0e+00 | FS943322 | small | 18 | 17q25 |
| *CA10* | 1.1 | 691 | 4.4e-136 | FS943323 | small | 18 | 17q21.33 |
| *CCT6A* | 1.0 | 601 | 8.2e-53 | FS943324 | small | 19 | 7p11.2 |
| *ENSGALESTG00000010266*^§^ | 1.0 | 610 | 3.2e-56 | FS943325 | small | 19 | no |
| *KCNG1* | 1.3 | 655 | 0.0e+00 | FS943326 | small | 20 | 20q13 |
| *CAPZB* | 1.1 | 634 | 1.1e-110 | FS943327 | small | 21 | 1p36.1 |
| *TMEM50A* | 1.0 | 635 | 3.1e-72 | FS943328 | small | 23 | 1p36.11 |
| *SFPQ* | 1.3 | 643 | 4.2e-80 | FS943329 | small | 23 | 1p34.3 |
| *RAP1A* | 1.5 | 712 | 3.1e-106 | FS943330 | small | 26 | 1p13.3 |
| *CAPZA1* | 2.4 | 707 | 2.9e-105 | FS943331 | small | 26 | 1p13.2 |
| *RPL10A* | 0.9 | 509 | 1.2e-82 | FS943332 | small | 26 | 6p21.31 |
| *FKBP5* | 1.3 | 686 | 9.9e-115 | FS943333 | small | 26 | 6p21.31 |
| *OAZ1* | 0.8 | 609 | 9.9e-156 | FS943334 | small | 28 | 19p13.3 |
| *FAM32A* | 1.0 | 661 | 8.9e-56 | FS943335 | small | 28 | 19pter-p13.3 |
| ^*^Human gene symbol. ^†^E-values of reptile homologues versus chicken genes obtained with the BLAST and/or TBLASTX program of Ensembl (retrieved in March 2012). ^‡^Chromosomal locations of chicken and human homologues obtained with the BLASTN programs of Ensembl and/or NCBI (retrieved in March 2012). un, unknown chromosomal location. no, no homologues were found.  ^§^Gene symbol is not available, and its ortholog has not been determined yet. ^¶^Genes mapped in our previous study [1].  References  1. Kawai A, Nishida-Umehara C, Ishijima J, Tsuda Y, Ota H, et al. (2007) Different origins of bird and reptile sex chromosomes inferred from comparative mapping of chicken Z-linked genes. Cytogenet Genome Res 117: 92–102. | | | | | | | |
