# Supplementary material for: Inference of the Protokaryotypes of Amniotes and Tetrapods and the Evolutionary Processes of Microchromosomes from Comparative Gene Mapping
Source: PLoS One. 2012 Dec 31;7(12):e53027. doi: 10.1371/journal.pone.0053027 (PMC3534110; doi:10.1371/journal.pone.0053027)
Supplement: Table S3 — List of 140 genes that were localized to chromosomes of X. tropicalis. (DOC) [file pone.0053027.s008.doc]

| **Table S3** |  |  |  |  |  |  |
| --- | --- | --- | --- | --- | --- | --- |
| List of 140 genes that were localized to chromosomes of *Xenopus tropicalis.* | | | |  |  |  |
| Gene symbol^*^ | Clone no.^†^ or Accession no. | Scaffold no.^‡^ | Chromosomal　location | | |  |
|  |  |  | *X. tropicalis* | chicken^§^ | human^§^ |  |
| *KDM3A* | XL413k20ex | Scaffold GL172916.1 | 1p | 4q | 2p11.2 |  |
| *CCT7* | XL334d10ex | Scaffold GL172834.1 | 1p | 4q | 2p13.2 |  |
| *MSX1* | XL477f10ex | Scaffold GL173077.1 | 1p | 4q | 4p16.3-p16.1 |  |
| *ACSL1* | XL318g05ex | Scaffold GL172726.1 | 1p | 4q | 4q34-q35 |  |
| *PCDH10* | XL003c24 | Scaffold GL172652.1 | 1p | 4q | 4q28.3 |  |
| *RAP1GDS1* | XL188b16 | Scaffold GL172870.1 | 1p | 4q | 4q23-q25 |  |
| *EEF2* | XL470l07ex | Scaffold GL173404.1 | 1q | 28 | 19pter-q12 |  |
| *AP1M1* | XL460b16ex | Scaffold GL173046.1 | 1q | 28 | 19p13.12 |  |
| *PLIN2* | XL259d05ex | Scaffold GL172798.1 | 1q | Zq | 9p22.1 |  |
| *DMRT1*^¶^ | AB201112 | Scaffold GL172794.1 | 1q | Zq | 9p24.3 |  |
| *NF2* | XL085b04 | Scaffold GL172727.1 | 1q | 15 | 22q12.2 |  |
| *PITPNB* | XL090i02 | Scaffold GL173872.1 | 1q | 15 | 22q12.1 |  |
| *TOP3B* | XL306l05ex | Scaffold GL172648.1 | 1q | 15 | 22q11.22 |  |
| *SNX2* | XL065o20 | Scaffold GL172643.1 | 1q | Zq | 5q23 |  |
| *APC* | XL064m16 | Scaffold GL172846.1 | 1q | Zq | 5q21-q22 |  |
| *TTC37* | XL071e03 | Scaffold GL172694.1 | 1q | Zq | 5q15 |  |
| *DEPDC1B* | XL220a24 | Scaffold GL172985.1 | 1q | Zp | 5q12.1 |  |
| *ZFR* | XL452j10ex | Scaffold GL172864.1 | 1q | Zp | 5p13.3 |  |
| *ATP5A1* | XL274m08ex | Scaffold GL172853.1 | 1q | Zp | 18q12-q21 |  |
| *POU2F1* | XL164e23 | Scaffold GL173866.1 | 2p | 1q | 1q22-q23 |  |
| *BAZ1B*|| | BC072944 | Scaffold GL172700.1 | 2p | 19 | 7q11.23 |  |
| *EIF2S3* | XL408e08ex | Scaffold GL172771.1 | 2p | 1q | Xp22.2-p22.1 |  |
| *ATP1B1* | XL216n10 | Scaffold GL172805.1 | 2p | 1q | 1q24 |  |
| *TSPAN7* | XL095n03 | Scaffold GL172671.1 | 2p | 1q | Xp11.4 |  |
| *WNT2B* | XL014m21 | Scaffold GL172654.1 | 2p | 26 | 1p13 |  |
| *CEPT1* | XL083p12 | Scaffold GL172830.1 | 2p | 26 | 1p13.3 |  |
| *TADA2L*|| | NM_001016651 | Scaffold GL173152.1 | 2q | 19 | 17q12-q21 |  |
| *TAF8* | XL003a04 | Scaffold GL172677.1 | 2q | 26 | 6p21.1 |  |
| *EPB41* | XL284j18ex | Scaffold GL173128.1 | 2q | 23 | 1p33-p32 |  |
| *PUM1* | XL227l19ex | Scaffold GL173114.1 | 2q | 23 | 1p35.2 |  |
| *YARS* | XL197i03 | Scaffold GL173188.1 | 2q | 23 | 1p35.1 |  |
| *MDH2* | XL165g11 | Scaffold GL172708.1 | 2q | 19 | 7cen-q22 |  |
| *CCT6A* | XL443e05ex | Scaffold GL172807.1 | 2q | 19 | 7p11.2 |  |
| *ING1* | XL060b15 | Scaffold GL172678.1 | 2q | 1q | 13q34 |  |
| *SPRY2* | XL211j19 | Scaffold GL172649.1 | 2q | 1q | 13q31.1 |  |
| *MTRF1* | XL181g24 | Scaffold GL172972.1 | 2q | 1q | 13q14.1-q14.3 |  |
| *LARP4* | XL014a12 | Scaffold GL172703.1 | 2q | un | 12q13.12 |  |
| *RACGAP1* | XL209i12 | Scaffold GL172803.1 | 2q | un | 12q13.12 |  |
| *SERPINH1* | XL217i12 | Scaffold GL172913.1 | 2q | 1q | 11q13.5 |  |
| *RAB6A* | XL038d18 | Scaffold GL174001.1 | 2q | 1q | 11q13.3 |  |
| *PPFIBP1* | XL062p09 | Scaffold GL173841.1 | 3p | 1p | 12p11.23-p11.22 |  |
| *PPARA* | XL510n05ex | Scaffold GL173303.1 | 3p | 1p | 22q13.31 |  |
| *IK* | XL168o22 | Scaffold GL173204.1 | 3q | 13 | 5q31.3 |  |
| *SGCD* | XL082j24 | no | 3q | 13 | 5q33-q34 |  |
| *RUFY1* | XL024b24 | Scaffold GL172949.1 | 3q | 13 | 5q35.3 |  |
| *CSNK1A1* | XL221g09ex | Scaffold GL173083.1 | 3q | 13 | 5q32 |  |
| *EGR1* | XL212p15 | Scaffold GL172879.1 | 3q | 13 | 5q31.1 |  |
| *METAP2* | XL214k05 | Scaffold GL173713.1 | 3q | 1p | 12q22 |  |
| *GNAI1* | XL239i19ex | Scaffold GL172681.1 | 3q | 1p | 7q21 |  |
| *NET1* | XL227m13ex | Scaffold GL172647.1 | 3q | 1p | 10p15 |  |
| *TRIP4* | XL142j06 | Scaffold GL173053.1 | 3q | 10 | 15q22.31 |  |
| *NPTN* | XL214k06 | Scaffold GL172739.1 | 3q | 10 | 15q22 |  |
| *CYP19A1*^¶^ | BC079750 | Scaffold GL172839.1 | 3q | 10 | 15q21.1 |  |
| *GLCE* | XL210o14 | Scaffold GL172676.1 | 3q | 10 | 15q23 |  |
| *GFPT1* | XL445a17ex | Scaffold GL172779.1 | 3q | 22 | 2p13 |  |
| *SLC20A1* | XL164n10 | Scaffold GL172666.1 | 3q | 22 | 2q11-q14 |  |
| *XPO7* | XL214l11 | Scaffold GL172670.1 | 3q | 22 | 8p21 |  |
| *SCUBE2* | XL210o03 | Scaffold GL173751.1 | 4p | 5q | 11p15.3 |  |
| *WT1*^¶^ | D82051 | Scaffold GL173035.1 | 4p | 5p | 11p13 |  |
| *EDC4* | XL473f03ex | Scaffold GL172782.1 | 4p | 11 | 16q22.1 |  |
| *KARS* | XL480m02ex | Scaffold GL172756.1 | 4q | 11 | 16q23-q24 |  |
| *PDCD5* | XL512n07ex | Scaffold GL172730.1 | 4q | 11 | 19q12-q13.1 |  |
| *WLS* | XL066b03 | Scaffold GL173067.1 | 4q | 8q | 1p31.3 |  |
| *TMEM48* | XL260h10ex | Scaffold GL172637.1 | 4q | 8q | 1p32.3 |  |
| *UAP1* | XL086p04 | Scaffold GL172721.1 | 4q | 8p | 1q23.3 |  |
| *NR2C2* | XL190f24 | Scaffold GL172989.1 | 4q | 12 | 3p25 |  |
| *ALAS1* | XL051o04 | Scaffold GL173517.1 | 4q | 12 | 3p21.1 |  |
| *TKT* | XL436o06ex | Scaffold GL174118.1 | 4q | 12 | 3p14.3 |  |
| *NVL* | XL479c13ex | Scaffold GL173164.1 | 5p | 3q | 1q41-q42.2 |  |
| *XPO1* | XL294p07ex | Scaffold GL173131.1 | 5p | 3p | 2p16 |  |
| *CEBPZ* | XL039l04 | Scaffold GL173606.1 | 5p | 3q | 2p22.2 |  |
| *SLC2A12* | XL036o21 | Scaffold GL172808.1 | 5q | 3q | 6q23.2 |  |
| *AMD1* | XL084d10 | Scaffold GL172886.1 | 5q | 3q | 6q21-q22 |  |
| *LMBRD1* | XL255c05ex | Scaffold GL172837.1 | 5q | 3q | 6q13 |  |
| *KPNA4* | XL254f07ex | Scaffold GL172686.1 | 5q | 9 | 3q25.33 |  |
| *TRIP12* | XL204l01 | Scaffold GL172691.1 | 5q | 9 | 2q36.3 |  |
| *AMOTL2* | XL239e08ex | Scaffold GL172765.1 | 5q | 9 | 3q21-q22 |  |
| *SAP130* | XL282g08ex | Scaffold GL172860.1 | 5q | 9 | 2q14.3 |  |
| *GATA4* | XL039m17 | Scaffold GL172699.1 | 5q | 3q | 8p23.1-p22 |  |
| *ABCF2* | XL012l18 | Scaffold GL173706.1 | 6p | 2p | 7q36 |  |
| *BICC1* | XL162n21 | Scaffold GL172899.1 | 6p | 6 | 10q21.1 |  |
| *WAC* | XL075d07 | Scaffold GL172683.1 | 6p | 2p | 10p11.2 |  |
| *HOXA1* | XL095h23 | Scaffold GL172692.1 | 6p | 2p | 7p15.3 |  |
| *CTNNB1* | XL480g03ex | Scaffold GL173301.1 | 6p | 2p | 3p21 |  |
| *PDIA4* | XL004n24 | Scaffold GL173401.1 | 6p | 2q | 7q35 |  |
| *GRB10* | XL027l13 | Scaffold GL172693.1 | 6p | 2q | 7p12-p11.2 |  |
| *CCT5* | XL515n08ex | Scaffold GL172641.1 | 6p | 2q | 5p15.2 |  |
| *SERPINB6* | XL502p18ex | Scaffold GL172731.1 | 6q | 2q | 6p25 |  |
| *APCDD1* | XL055h12 | Scaffold GL178258.1 | 6q | 2q | 18p11.22 |  |
| *LRRCC1* | XL033o08 | Scaffold GL173088.1 | 6q | 2q | 8q21.2 |  |
| *YWHAZ* | XL445d16ex | Scaffold GL172639.1 | 6q | 2q | 8q23.1 |  |
| *EEF1D* | XL013c21 | Scaffold GL173373.1 | 6q | 2q | 8q24.3 |  |
| *SEC23IP* | XL151i10 | Scaffold GL173241.1 | 7p | 6 | 10q25-q26 |  |
| *USP5* | XL016m06 | Scaffold GL173371.1 | 7p | 1q | 12p13 |  |
| *ZRANB1* | XL027a17 | Scaffold GL172952.1 | 7p | 6 | 10q26.13 |  |
| *CYP17A1*^¶^ | AF325435 | Scaffold GL173037.1 | 7p | 6 | 10q24 |  |
| *PGAM1* | XL323m11ex | Scaffold GL173153.1 | 7p | 6 | 10q25.3 |  |
| *GOT1* | XL151j11 | Scaffold GL172907.1 | 7p | 6 | 10q24 |  |
| *TUBGCP2* | XL008b12 | Scaffold GL173234.1 | 7p | 6 | 10q26.3 |  |
| *ACAD8* | XL226j18ex | Scaffold GL172967.1 | 7q | 24 | 11q25 |  |
| *SLC37A2* | XL082p15 | Scaffold GL172933.1 | 7q | 24 | 11q24.2 |  |
| *ENO1* | XL095b23 | Scaffold GL172843.1 | 7q | 21 | 1p36.3-p36.2 |  |
| *ZW10* | XL046l12 | Scaffold GL172954.1 | 7q | 24 | 11q23.2 |  |
| *ERRFI1* | XL006h11 | Scaffold GL172929.1 | 7q | 21 | 1p36 |  |
| *DDOST* | XL005p17 | Scaffold GL173831.1 | 7q | 21 | 1p36.1 |  |
| *GPR107* | XL305g08ex | Scaffold GL172827.1 | 8p | 17 | 9q34.11 |  |
| *GSN* | XL300b05ex | Scaffold GL173389.1 | 8p | 17 | 9q33 |  |
| *NR5A1*^¶^ | AB273177 | Scaffold GL173041.1 | 8q | 17 | 9q33 |  |
| *AR*^¶^ | U67129 | Scaffold GL173497.1 | 8q | 4p | Xq11.2-q12 |  |
| *OGT* | XL087m04 | Scaffold GL172753.1 | 8q | 4p | Xq13 |  |
| *FMR1* | XL022n18 | Scaffold GL172738.1 | 8q | 4p | Xq27.3 |  |
| *PGK1* | XL072d06 | Scaffold GL173229.1 | 8q | 4p | Xq13 |  |
| *SOX3*^¶^ | – | Scaffold GL172698.1 | 8q | 4p | Xq27.1 |  |
| *FGF13* | XL081g06 | Scaffold GL172667.1 | 8q | 4p | Xq27 |  |
| *BRD2* | XL303i04ex | Scaffold GL173531.1 | 8q | 16 | 6p21.3 |  |
| *CBX6* | XL023a17 | Scaffold GL172705.1 | 8q | 1p | 22q13.1 |  |
| *PSMC1* | XL318k24ex | Scaffold GL172685.1 | 8q | 5q | 14q32.11 |  |
| *PSMC6* | XL322j19ex | Scaffold GL172883.1 | 8q | 5q | 14q22.1 |  |
| *PAPOLA* | XL035j18 | Scaffold GL172821.1 | 8q | 5q | 14q32.31 |  |
| *ACTN1* | XL286g19ex | Scaffold GL172781.1 | 8q | 5q | 14q22-q24 |  |
| *RAD51* | XL186a11 | Scaffold GL173263.1 | 8q | 5p | 15q15.1 |  |
| *GPHN* | XL071a10 | Scaffold GL172909.1 | 8q | 5q | 14q23.3 |  |
| *VPS45* | XL316m01ex | Scaffold GL173012.1 | 8q | 25 | 1q21.2 |  |
| *COPA* | XL263c24ex | Scaffold GL172915.1 | 8q | 25 | 1q23-q25 |  |
| *UQCRC2* | XL016d01 | Scaffold GL172650.1 | 9p | 14 | 16p12 |  |
| *NOMO3* | XL271k19ex | Scaffold GL172657.1 | 9p | 14 | 16p13 |  |
| *SF3B1* | XL298m06ex | Scaffold GL172638.1 | 9q | 7q | 2q33.1 |  |
| *NDUFS1* | XL034k12 | Scaffold GL172844.1 | 9q | 7q | 2q33-q34 |  |
| *FN1* | XL338p06ex | Scaffold GL172651.1 | 9q | 7p | 2q34 |  |
| *ZEB2* | XL207j23 | Scaffold GL172868.1 | 9q | 7q | 2q22 |  |
| *BMP7* | XL056l08 | Scaffold GL173417.1 | 10p | 20 | 20q13 |  |
| *ACLY* | XL322c19ex | Scaffold GL173076.1 | 10p | 27 | 17q12-q21 |  |
| *KAT7* | XL035p09 | Scaffold GL172801.1 | 10p | 27 | 17q21.32 |  |
| *EFTUD2* | XL300a14ex | Scaffold GL172791.1 | 10p | 27 | 17q21.31 |  |
| *SOX9*^¶^ | AB439583 | Scaffold GL172812.1 | 10q | 18 | 17q24.3-q25.1 |  |
| *P4HB* | XL512o03ex | Scaffold GL172918.1 | 10q | 18 | 17q25 |  |
| *STAU1* | XL175g01 | Scaffold GL172684.1 | 10q | 20 | 20q13.1 |  |
| *SRSF6* | XL473a23ex | Scaffold GL172674.1 | 10q | 20 | 20q12-q13.1 |  |
| *DDX5* | XL519l13ex | Scaffold GL173616.1 | 10q | 18 | 17q21 |  |
| *NARF* | XL210h06 | Scaffold GL172814.1 | 10q | 18 | 17q25.3 |  |
| ^*^Human gene symbol. ^†^Clone numbers of *X. laevis* EST clones used for mapping, which were selected from a web data catalogue of NIBB/NIG/NBRP *Xenopus laevis* EST project (XDB3, http://xenopus.nibb.ac.jp/) using the nucleotide sequences of *X. tropicalis* homologs, which were subjected to a search with the BLASTN program of Ensembl. The fragment sizes of all *X. laevis* EST clones were more than 1.5 kb. ^‡^Scaffold numbers of *X. tropicalis* obtained with the BLASTN program of the Ensembl (retrieved in March 2012). no, no homologues were found. We confirmed that these genes used for mapping were each located in different scaffolds of *X. tropicalis* using the genome map of *X. tropicalis* (the Ensembl Xenopus tropicalis Genome Browser, http://www.ensembl.org/Xenopus_tropicalis). ^§^Chromosomal locations of chicken and human homologues obtained with the BLASTN programs of Ensembl and/or NCBI (retrieved in March 2012). un, unknown chromosomal location. no, no homologues were found.  ^¶^Genes mapped in our previous study [1]. *X. laevis* homologue of *SOX3* was isolated by Koyano et al. [2]. ||We molecularly cloned partial cDNA fragments of the *X. laevis* *BAZ1B* and *X. tropicalis* *TADA2A* genes on the basis of the nucleotide sequences deposited under their accession numbers in the NCBI database.  References  1. Uno Y, Nishida C, Yoshimoto S, Ito M, Oshima Y, et al. (2008) Diversity in the origins of sex chromosomes in anurans inferred from comparative mapping of sexual differentiation genes for three species of the Raninae and Xenopodinae. Chromosome Res 16: 999–1011.  2. Koyano S, Ito M, Takamatsu N, Takiguchi S, Shiba T (1997) The *Xenopus Sox3* gene expressed in oocytes of early stages. Gene 188: 101–107. | | | | | | |
|  |  |  |  |  |  |  |
